# Dynamic Evolution of Pathogenicity Revealed by Sequencing and Comparative Genomics of 19 *Pseudomonas syringae* Isolates

**DOI:** 10.1371/journal.ppat.1002132

**Published:** 2011-07-14

**Authors:** David A. Baltrus, Marc T. Nishimura, Artur Romanchuk, Jeff H. Chang, M. Shahid Mukhtar, Karen Cherkis, Jeff Roach, Sarah R. Grant, Corbin D. Jones, Jeffery L. Dangl

**Affiliations:** 1 Department of Biology, University of North Carolina at Chapel Hill, Chapel Hill, North Carolina, United States of America; 2 Research Computing Center, University of North Carolina at Chapel Hill, Chapel Hill, North Carolina, United States of America; 3 Curriculum in Genetics and Molecular Biology, University of North Carolina at Chapel Hill, Chapel Hill, North Carolina, United States of America; 4 Carolina Center for Genome Sciences, University of North Carolina at Chapel Hill, Chapel Hill, North Carolina, United States of America; 5 Department of Microbiology and Immunology, University of North Carolina at Chapel Hill, Chapel Hill, North Carolina, United States of America; University of Toronto, Canada

## Abstract

Closely related pathogens may differ dramatically in host range, but the molecular, genetic, and evolutionary basis for these differences remains unclear. In many Gram- negative bacteria, including the phytopathogen *Pseudomonas syringae*, type III effectors (TTEs) are essential for pathogenicity, instrumental in structuring host range, and exhibit wide diversity between strains. To capture the dynamic nature of virulence gene repertoires across *P. syringae*, we screened 11 diverse strains for novel TTE families and coupled this nearly saturating screen with the sequencing and assembly of 14 phylogenetically diverse isolates from a broad collection of diseased host plants. TTE repertoires vary dramatically in size and content across all *P. syringae* clades; surprisingly few TTEs are conserved and present in all strains. Those that are likely provide basal requirements for pathogenicity. We demonstrate that functional divergence within one conserved locus, *hopM1*, leads to dramatic differences in pathogenicity, and we demonstrate that phylogenetics-informed mutagenesis can be used to identify functionally critical residues of TTEs. The dynamism of the TTE repertoire is mirrored by diversity in pathways affecting the synthesis of secreted phytotoxins, highlighting the likely role of both types of virulence factors in determination of host range. We used these 14 draft genome sequences, plus five additional genome sequences previously reported, to identify the core genome for *P. syringae* and we compared this core to that of two closely related non-pathogenic pseudomonad species. These data revealed the recent acquisition of a 1 Mb megaplasmid by a sub-clade of cucumber pathogens. This megaplasmid encodes a type IV secretion system and a diverse set of unknown proteins, which dramatically increases both the genomic content of these strains and the pan-genome of the species.

## Author Summary

Breakthroughs in genomics have unleashed a new suite of tools for studying the genetic bases of phenotypic differences across diverse bacterial isolates. Here, we analyze 19 genomes of *P. syringae*, a pathogen of many crop species, to reveal the genetic changes underlying differences in virulence across host plants ranging from rice to maple trees. Surprisingly, a pair of strains diverged dramatically via the acquisition of a 1 Mb megaplasmid, which constitutes roughly 14% of the genome. Novel plasmids and horizontal genetic exchange have contributed extensively to species-wide diversification. Type III effector proteins are essential for pathogenicity, exhibit wide diversity between strains and are present in distinct higher-level patterns across the species. Furthermore, we use sequence comparisons within an evolutionary context to identify functional changes in multiple virulence genes. Overall, our data provide a unique overview of evolutionary pressures within *P. syringae* and an important resource for the phytopathogen research community.

## Introduction

*Pseudomonas syringae* is a Gram-negative bacterial phytopathogen responsible for worldwide disease on many crop species [1]. Despite a collectively broad pathogenic range for the species, individual isolates of *P. syringae* display pathogenic potential on a limited set of plant species and either elicit immune responses, or simply fail to thrive on alternative species [2]–[4]. In addition to disease outbreaks, strains can be isolated as epiphytes from non-diseased plants as well as from multiple phases of the water cycle [5], [6]. How species with varied lifestyles like *P. syringae* maintain the genomic flexibility required to survive across this broad range of ecologies is not known. It remains particularly unclear how evolutionary forces shape the pan-genome of this species, especially virulence-related genes.

The host ranges of many *P. syringae* isolates or pathovars have not been thoroughly characterized. Research has largely focused on identifying the molecular basis of pathogenesis across three divergent strains with finished genome sequences and investigation of virulence mechanisms for a smattering of strains on a limited number of hosts [7]. These studies have shown that a Type III secretion system (TTSS), which acts like a molecular syringe to translocate a suite of type III effector (TTE) proteins into plant cells, is a key virulence determinant [7]–[9]. Once inside the plant cell, TTEs promote pathogenesis by disrupting and suppressing host defense responses at multiple levels [10]–[13]. TTEs can also be recognized by plant disease resistance proteins and recognition of a single effector is sufficient to trigger successful host immune response. However, the virulence functions of many TTEs are redundant, making these phenotypes potentially robust to host-mediated selection against single TTE genes [14]. Thus, host range is structured by the totality of a strain's TTE repertoire.

A high degree of divergence among commonly investigated isolates makes it nearly impossible to pinpoint all the changes that lead to host differentiation or specialization at the present time. As a result, key questions, such as what determines the overall plasticity of host range, remain unanswered. Deep sampling of diverse genomes within a phylogenetic framework can reveal general evolutionary trends indicative of changes in lifestyle and allow for the identification of genetic changes that differentiate between strains that have recently undergone host range shifts [3], [15].

Isolates of *P. syringae* are subdivided into approximately 50 pathovars based upon host range and comparison with type strains [16]. These are further subdivided into races based upon differential ability among strains within a pathovar to grow and cause disease across host genotypes [17]. Recent multilocus sequence typing (MLST) segregated *P. syringae* pathovars into at least 5 distinct phylogenetic clades [6], [16], [18], which largely mirror 9 genomospecies based on DNA hybridization [19], [20]. While the selection pressures determining host range may be similar throughout the species, there has simply not been deep enough phenotypic sampling or sequencing of genomes across the species to uncover trends indicating evolutionary differentiation among the clades [4].

Multiple screens, primarily within the three *P. syringae* strains with completely sequenced ‘gold standard’ genomes [3], [21]–[30], have suggested that the number of TTEs per genome ranges from ∼20 to 33, with the total number of validated TTE protein families ∼50. However, efforts to catalogue the TTE repertoires from various strains often fall short of capturing a complete picture. For instance, false negatives occur from lack of saturation in functional TTE screens or because sequence divergence confounds hybridization based methods. False positives also occur with hybridization methods when the gene sequences are present but contain frame-shifts or disruptions, or are only partial matches to the known TTE genes (i.e. chimeras [31]). Most of these limitations are obviated by whole genome sequences, especially when combined with orthogonal functional methods to validate candidates as TTEs.

The TTSS is not the sole determinant of virulence and host range for *P. syringae*; coordination of host physiological responses and metabolic pathways is also necessary for pathogen growth within host tissue [32]. Phytotoxins, which can be coordinately regulated with the TTSS, but secreted independently from the TTEs [33], can disrupt host metabolism or act as mimics of plant hormones. Hence, they may replace or complement virulence functions of TTEs [34]. Indeed, manipulation of stomatal function by coronatine, a structural mimic of the plant hormone jasmonic acid, is essential for invasion of *A. thaliana* leaves by *P. syringae pv. tomato* (*Pto*) DC3000. However, coronatine also possesses independent virulence functions during the colonization of roots [35]. Therefore, pathogenesis of *P. syringae* on any given plant host species, results from both the absence of avirulence factors (an operational definition of TTEs that activate a host immune receptor) and the presence of multiple virulence factors acting coordinately to promote disease and to suppress host immune responses [4], [7], [13].

To date, genomics studies in *P. syringae* suggest that virulence mechanisms within this species are evolutionarily dynamic and have experienced strong selective pressures [3], [36]. Complete genome sequences exist for three phylogenetically diverse *P. syringae* isolates representing MLST groups I, II, and III (*Pto* DC3000, *P. syringae pv. syringae* B728a (*Psy*), and *P. syringae pv. phaseolicola* 1448a (*Pph*), respectively; [26]–[28]. Recently, additional draft genome sequences were generated by either Roche/454 or Illumina sequencing technologies (for *Pto* T1, group I; *Pta* ATCC11528, pathovar *tabaci*; *Psv* NCPP3335, pathovar *savastanoi*; and multiple strains from pathovars *aesculi* and *glycinea*, all group III; [30], [37]–[40]), or a hybrid genome assembly pipeline utilizing both Illumina and Roche sequencing technologies (*Por* 1_6, pathovar *oryzae*, group IV; [41]). The genomes of these *P. syringae* strains differ dramatically in gene and plasmid content and in the presence/absence of many virulence-related genes. Given that these strains represent only a fraction of the known diversity within *Pseudomonas* isolates, much of the phylogenetic, ecological, and host diversity for this plant pathogen remains unexplored.

We provide a phylogenetically comprehensive genomic view of *P. syringae* with a focus on TTE repertoire evolution. We analyzed data from draft or complete genome sequences of 19 diverse isolates, including 14 new draft genome sequences. We couple these genome sequences with a functional screen to identify new TTE families from diverse strains. The TTE content within these strains, as well as the presence of other known pathogenesis-related genes, is volatile. We show that cost-efficient genome sequencing placed within a phylogenetic context provides a thorough and unique viewpoint into *P. syringae* evolution and sheds light on previously unrecognized evolutionary patterns and structural diversity for this important plant pathogen.

## Results

### High Quality Draft Genome Sequences for Phylogenetically Diverse Strains of *P. syringae*

We employed a hybrid approach [41] utilizing reads from both Illumina and 454 platforms to generate draft genome sequences for 14 phylogenetically diverse strains of *P. syringae* (Table 1). These draft genomes are each contained on 32 to 222 scaffolds with the N50 value at 81,010 bp (e.g. half of the total sequenced genome, calculated by summing the lengths of all contigs and scaffolds within a given strain, is found in scaffolds 81,010 bp or greater). Although each genome assembly varies slightly, the size distribution of contigs and scaffolds (Figure S1) is equivalent to what we previously described for our hybrid assembly of re-sequenced *Pto* DC3000 compared to the published sequence of the same strain [26], [41].

**Table 1:** Draft genome sequencing summary for 14 phylogenetically divergent P. syringae strains.

Identifier	*Pgy* R4	*Pmo*	*Pta*	*Pae*	*Pla* 107	*Cit7*	*Pac*	*Ppi* R6	*Pja*	*Ptt*	*Pma*	*Pla* 106	*Pmp*	*Pan*
Pathovar	*glycinea*	*mori*	*tabaci*	*aesculi*	*lachrymans*	*NA*	*aceris*	*pisi*	*japonica*	*aptata*	*maculicola*	*lachrymans*	*morsprunorum*	*actinidiae*
Strain	A29-2	MAFF 301020	ATCC11528	0893_23	MAFF									
301315	Cit7	MAFF 302273PT	1704B	MAFF 301072 PT	DSM50252	ES4326	MAFF 302278PT	MAFF 302280PT	MAFF302091					
Illumina Reads	4,251,697	7,592,243	5,030,953	15,848,526	8,360,650	5,319,177	6,897,439	4,637,560	5,122,223	5,446,792	14,975,328	8,177,882	16,637,970	10,336,469
Illumina Bases	153,061,092	265,728,505	181,114,308	554,698,410	641,057,438	191,490,372	241,410,365	166,952,160	184,400,028	196,084,512	524,136,480	294,403,752	1,247,847,750	361,776,415
454 Reads	432,292	162,625	131,130	130,109	126,287	149,557	155,239	285,725	375,650	144,250	300,835	345,257	141,417	299,744
454 Bases	30,418,492	24,154,020	19,726,747	19,173,310	20,116,024	22,337,212	22,063,131	22,457,735	24,292,249	21,590,755	23,408,095	26,294,110	21,665,692	22,364,744
454 Paired Ends	116,271	51,096	39,842	41,071	33,455	46,380	51,113	104,112	108,436	44,421	110,879	84,301	42,284	112,016
# Contigs	4,430	3,414	1,613	915	791	2,655	1,179	5,099	4,661	3,776	878	798	969	941
Contigs N50	3,723	5,634	16,098	16,806	22,550	6,862	12,409	3,003	4,021	4,753	17,222	15,738	15,161	14,086
# Scaffolds	109	70	32	139	222	57	60	170	60	53	44	90	69	138
Scaffolds N50	111,252	203,999	344,662	81,010	129,539	399,070	176,541	83,352	181,972	165,542	340,783	135,618	175,394	69,188
NCBI Accession # <$>\raster="rg1"<$>	ADWY	AEAG	AEAP	AEAD	AEAF*	AEAJ	AEAO	AEAI	AEAH	AEAN	AEAK	AEAM	AEAE	AEAL
Included in TTE Screen	Yes	Yes	No	No	Yes	No	Yes	Yes	No	Yes	*Pma* M4	Yes	Yes	Yes

^<$>\raster="rg1"<$>^All accession numbers are followed by 00000000.

*Accession # for the *Pla* 107 Mega Plasmid Sequence is CM000959.

**Figure 1:**
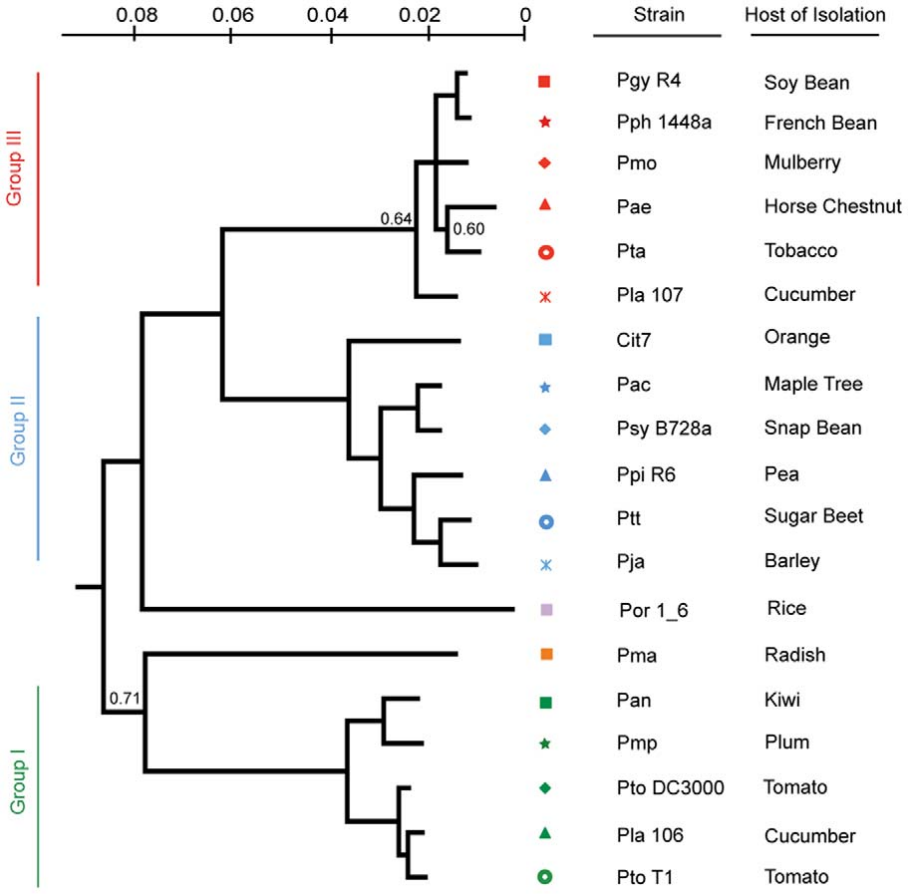
A Bayesian phylogeny of *P. syringae* strains with draft or complete genome sequences. Phylogenetic analysis of the 19 strains included in this study based on nucleotide sequence of seven conserved loci. Bayesian posterior probabilities are displayed on the phylogeny only at nodes where these values are <0.95. For these unresolved notes, we used an independent phylogenetic approach on another 324 genes that confirmed that this tree captures the evolutionary history of these nodes (methods; Figure S2). Each phylogenetic group as defined in [16] was assigned its own color to the left of the phylogeny and strains were assigned symbols; this color and marker scheme continues throughout the figures. In all cases but one (Cit7; leaf surface of healthy Orange tree [95]) strains were isolated from diseased host plants listed at right.

We created a Bayesian phylogeny for the sequenced strains using fragments based on the MLST loci used in [16], but extended as far in these gene sequences as was possible to align (Figure 1). We also built maximum likelihood phylogenies by concatenating 324 protein sequences from a subset of proteins present in all strains, after establishing orthology and producing amino acid alignments using a hidden Markov model (Figure S2), as well as individual phylogenies of these 324 protein sequences (Dataset S8). Our phylogenies are largely congruent with prior work, however, we find that the exact placement of *Por* and *Pma* and the resolution of the relationships between *Pmo*, *Pta*, *Pae*, and *Pla 107* (see Table 1 for strain key) are sensitive to the phylogenetic method used (data not shown). In cases of discrepancies between the tree inferred from MLST sequences, the tree from 324 concatenated sequences, and the individual protein trees, such as the placement of *Pae*, the second most probable protein tree invariably supported the topology inferred from MLST sequences (Figure S2, Dataset S8).

### Defining the *P. syringae* Core and Pan Genome

**Figure 2:**
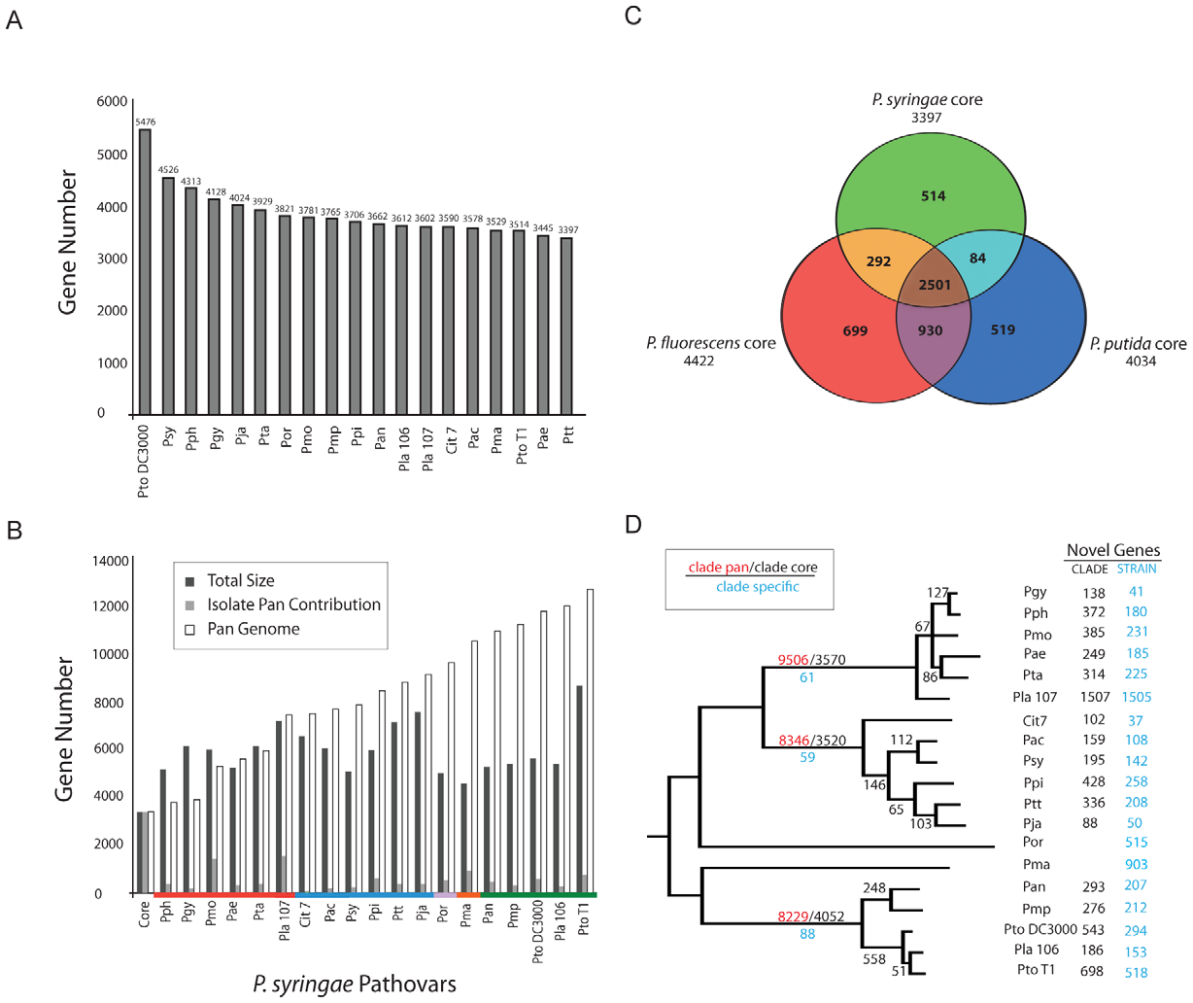
The core- and pan-genome of *P. syringae*. Collectively, *P. syringae* isolates share ∼50% of their ORFs with other pseudomonads. (**A**) The *P. syringae* core genome contains 3397 genes. (**B**) The *P. syringae* pan genome contains 12749 ORFs. (**C**) *P. syringae*, *P. fluorescens*, and *P. putida* share 2501 ORFs. *P. syringae* has the smallest core genome (3397) compared to *P. fluorescens* and *P. putida* (4422, 4034 respectively). *P. fluorescens* and *P. putida* share more genes with each other than either does with *P. syringae*. (**D**) Phylogenetic distribution of shared and clade/strain specific genes. Numbers on the earliest branch for each group indicate the size of the core (black) and pan (red) genomes for groups with multiple sequenced genomes (I, II, III), as well as the number of clade specific ORFs (blue, conserved within each group but absent from other groups). Internal branches display the number of ORFs gained, and shared by all genomes, after each branch bifurcation (see Methods). Numbers of ORFs within each genome absent from other strains within the relevant *P. syringae* group (black) and throughout the species (blue) are shown at the far right. Group I strains (including *Pto* DC3000) contain the largest number of shared ORFs and the smallest number of pan ORFs. *Pja* and *Pla* 107 have the smallest and the largest number of unique ORFs (88, 1507 respectively).

The core genome consists of those genes found in all sequenced genomes of a species. Following automated NCBI and manual Phylo-gene-boost annotation (Materials and Methods; Figure S3; Dataset S1, S2, S3, S4) and using a somewhat liberal approach to defining similarity (40% identity over 40% length), we defined a *P. syringae* core genome of 3,397 genes (Figure 2A). In contrast, the 12,749 genes of the pan genome are found only in subsets of strains (Figure 2B). We extended this analysis to include genomic information from multiple genome sequences of two related pseudomonad lineages: a plant-associated non-pathogenic bacterium (*P. fluorescens*; 3 genomes; [42], [43]) and a soil bacterium (*P. putida*; 4 genomes; [44], strains GB-1, F1, W619 unpublished but available at Genbank) (Figure 2C). A core of 2,501 genes was found within all isolates of all three of these pseudomonad lineages (Figure 2C; Figure S7). The 292 core genes shared between *P. syringae* and *P. fluorescens* that are not shared with *P. putida* are candidate plant-association loci and are enriched with genes predicted to be involved in protein localization and transport (Figure S8). There are 514 genes within the *P. syringae* core genome absent from the three-species core (Figure S9). These include a disproportionate number of metabolic regulators, protein localization and transport genes (Figure S9). The overall percentage of strain-specific genes, 5–10%, is fairly consistent across the *P. syringae* phylogeny (Figure 2D), the one exception being *Pla* 107. Roughly one in seven genes within this genome are strain-specific, and most of these are contained on a single megaplasmid (see below).

We analyzed the core and pan genomes for the three major clades of *P. syringae* (groups I, II, and III according to [16]) (Figure 2D; Dataset S5, S6, S7). Even though each group possesses similar levels of nucleotide sequence divergence, we found that group I strains have ∼500 more genes within their core genome than groups II and III (Figures 2D, S10). Since the number of sequenced isolates is smaller for group I, we performed bootstrapping analysis of the other two clades to show that the inflated core genome of the group I strains is robust to differences in the number of genome sequences sampled (data not shown).

### Plasmids or Megaplasmids Are Found in Most Pathovars

Current assembly methods for short read technologies are poor at assembling across repetitive regions. Thus, we investigated the presence of plasmids within these strains using a multi-phase approach based upon the presence of plasmid structural genes within the draft genomes, similarity of loci present within these suspected plasmids to known plasmids from the NCBI database, and an approximation of plasmid coverage using Illumina read depth. We find that 13 out of 15 draft genomes likely contain plasmids (Table S1, Figure S11), highlighting the importance of extra-chromosomal elements in the evolution of *P. syringae* [45].

The *Pla* 107 genome assembled into scaffolds representative of a typical chromosome, as well as a ∼1 Mb scaffold with approximately the same GC content and depth of Illumina read coverage as known chromosomal genes (Figure S11) but with little sequence homology to the other *P. syringae* genomes. We used PCR and Sanger-based sequencing to confirm that this large scaffold was circular (Figure S12A,B). Hence, *Pla* 107 contains a ∼1 Mb megaplasmid. PCR-based screening shows that this megaplasmid is present within a closely related strain (*Pla* N7512), but absent or significantly modified in two other closely related strains (*Pla* YM7902 and *Pla* YM8003; Figure S12C). Draft genome sequences of closely related outgroups (*Pmo*, *Pta*) also lack the megaplasmid. Both strains that contain this extra-chromosomal element grow more slowly *in planta* and on plates (Figure S12D,E). Since these four *Pla* strains possess nearly identical sequences at their MLST loci, this megaplasmid is a recent acquisition. Although this extra-chromosomal element encodes an astonishing fraction of hypothetical proteins according to the NCBI annotation (776 of 1080 genes), as well as 35 additional conserved but uncharacterized proteins, it also contains “housekeeping” genes highly similar to those in other *Pseudomonas* species, a potential type IV secretion system distantly related to the *Legionella* Dot/Icm system, and 38 additional tRNA loci (bringing the total in this strain from 47 to 85). Although many type IV secretion system related structural genes do appear to be present, tBLASTn searches using sequences of known effector proteins did not produce likely hits [46], [47]. We also searched both the Conserved Domain Database (CDD) and KEGG to identify potential biochemical pathways on the megaplasmid, but found that no complete pathways were present (Fig. S12F). The “housekeeping” genes do not appear to be essential as there are often *P. syringae* homologues found on the main chromosome. The recent acquisition of this megaplasmid could signal the potential for a dramatic ecological shift across these closely related strains.

### Identification of Eight New Type III Effector Families

**Figure 3:**
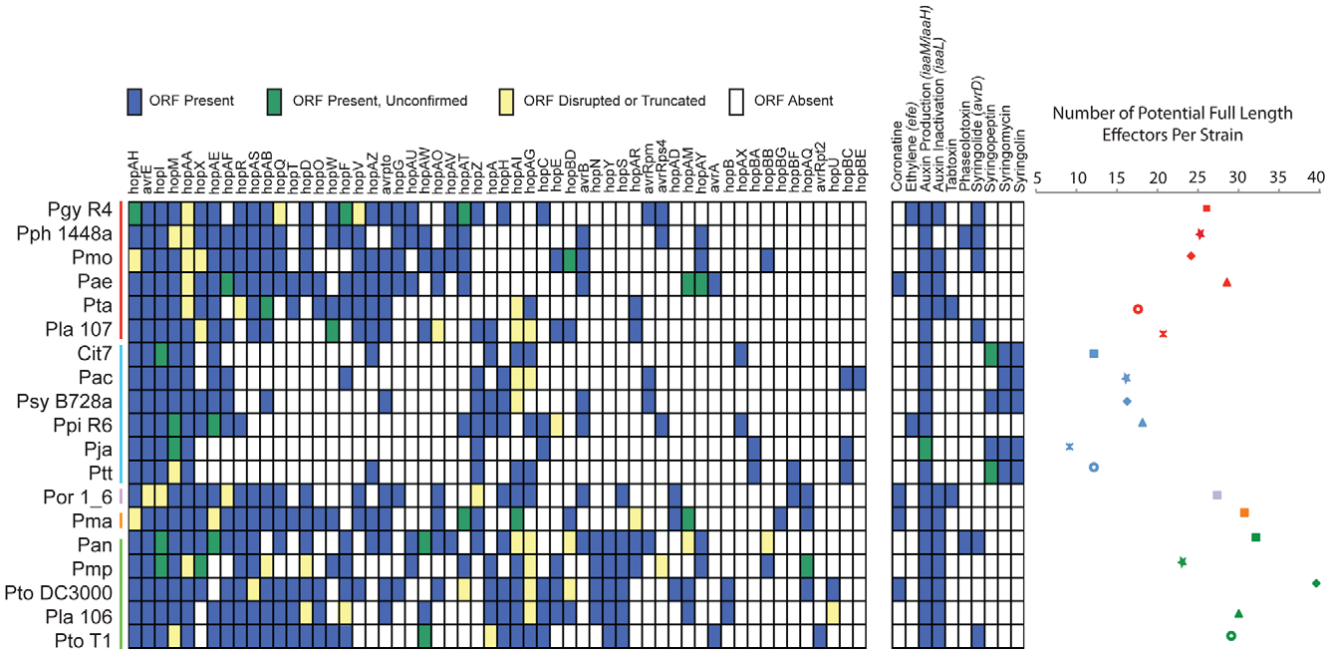
*P. syringae* isolates harbor extensive diversity in virulence gene repertoires. TTE, toxin, and plant hormone biosynthesis genes are listed across the top, *P. syringae* genomes, color-coded by phylogenetic group as in Figure 1. At the left, a blue box indicates presence of full-length ORFs or complete pathways within each genome. Green boxes indicate that genes or pathways are present by similarity searches, but the presence of full-length genes could not be verified by PCR, or the pathways are potentially incomplete. Yellow boxes indicate that genes are either significantly truncated or are disrupted by insertion sequence elements. White boxes indicate absence of genes or pathways from the strains based on homology searches. At the far right, the total number of potentially functional TTE proteins is shown for each genome and displayed according to the color-coded strain and group symbols shown in Figure 1.

The genomes from a subsample of the total sequenced isolates (*Pgy* R4, *Pmo*, *Pla* 107, *Pac*, *Ptt*, *Ppi* R6, *Pla* 106, *Pmo*, *Pan*, *Psy* B728a) were functionally screened for new TTE genes using a previously established method [23] based on the observation that many important virulence genes (and all known TTE) are regulated by the alternative sigma factor HrpL. Two additional strains that were not sequenced (*P. syringae* pv. *atrofaciens* DSM50255, and pv. *maculicola* M4) were also screened, and novel TTEs identified from these strains were included in all similarity searches (Dataset S9). We report the full results from all screened putative TTEs, as well as the type of locus identified in the screen (ORF only, or ORF including the putative *hrp*-box) in Dataset S9. From this screen, we identified and functionally validated by translocation assays members of eight new TTE families (Figure 3, Table 2). This increased the number of validated TTE families in *P. syringae* to 58 (not including *avrD*, defined according to unified nomenclature rules; [48]).

**Table 2:** Novel type three effectors.

hop name	Genbank accession	source strain	hrp box	BLASTp 10^−5^ cutoff	Strains containing
*hopAY1*	HM641785	*Pmo*	ggaactttttcttgcccgctaccac	Cysteine protease	*Pmp*, *Pan*, *Pae*, *Pmo*, *Pph*
*hopAZ1*	HM641786	*Pan*	ggaaccgcttttcaactgattgccac	Hypothetical (Pseudomonas)	*Pan*, *Pma*, *Ptt*, *Cit7*, *Pla* 107, *Pta*, *Pae*, *Pmo*, *Pgy*
*hopBA1*	HM641787	*Ptt*	ggaactgacaagccagtatgagccac	Hypothetical (Erwinia)	*Ptt*, *Pja*
*hopBB1*	HM641788	*Pmo*	ggaacttcaatagggtgtcgtaccac	C-terminus similar to *hopF2*	*Pmp*, *Pae*
*hopBC1*	HM641789	*Ptt*	ggaaccgtcttcgggacacggccac	Hypothetical (Erwinia)	*Ptt*, *Pja*, *Pac*
*hopBD1*	HM641790	*Pla* 107	ggaaccgatcgaggggttctgaccac	Hypothetical (Acidovorax)	*Pla* 106, *Pma*, *Pla* 107, *Pmo*
*hopBE1*	HM641791	*Pac*	ggaacccgatccatccgccgagccac	Hypothetical (Burkholderia)	*Pac*
*hopBF1*	HM641792	*Ptt*	ggaacccaactcactcaattcatcac	Hypothetical (Acidovorax)	*Por*, *Ptt*
*hopBG1*	HM641793	*Pma*	ggaaccgaatccatctcgagggccac	Hypotheical (Bradyrhizobium)	*Pma*

The characteristics of representative alleles for each new hop are listed. During analysis of this data, another group identified HopAY1 [96]. We include this TTE family in the table but not when describing the total number of TTE families discovered. BLASTp 10^−5^ cutoff column indicates the best hit for each allele in the NCBI NR database. The strains containing column indicates which of the sequence strains also contain alleles of these hop families. The translocation of representative alleles was verified, but consult Dataset S9 for translocation data for alleles from other genomes.

We are confident that there are now few undiscovered TTE families left to be found that are shared by a majority of these strains. First, we maximized phylogenetic diversity and screened four of the five major phylogenetic groups, using divergent strains within each of these groups. Second, our functional screen is close to saturation as measured by the recovery of known *hrpL*-regulated TTSS loci from the respective genomes at frequencies similar to previously published reports (Table S2; [23]).

We list instances where homologues of previously known TTE families were identified in the screened genomes (Dataset S9). In this file we also list instances where genes were identified as HrpL-regulated within the screen, but which were not translocated according to our tests. Since these genes are confirmed to be HrpL-regulated, and are therefore linked to the major pathogenic regulon in *P. syringae*, they could contribute to virulence in a translocation-independent way [29].

### Type III Effector and Toxin Content Varies Dramatically between Strains and Clades

We characterized the TTE content for each of the sequenced strains by similarity searches to all known *P. syringae* TTE (Figure 3; left). Our query list was generated by combining all previously identified *P. syringae* TTE with the eight new TTE families we identified (Materials and Methods; Dataset S9). We also acknowledge the limitation of our study in that TTEs and phytotoxin pathways may have been contained on plasmids or in other regions lost during sub-passaging of these strains. Overall, the total number of potential TTEs (defined as full length ORFs, confirmed for HrpL-induction, and with at least one family member translocated) varies dramatically between strains, from a minimum of 9 (*Pja*) to a maximum of 39 (*Pto* DC3000) (Figure 3; right). Furthermore, we find that the group II strains possess lower numbers of known TTEs on average than the other 4 groups. There are a total of five TTE families (*hopAA*, *avrE*, *hopM*, *hopI*, *hopAH*) present in some form (as either full length or truncated ORFs or disrupted by IS elements) within each of the sequenced strains. These represent the core TTEs found within all pathogenic *P. syringae* strains. These TTE are all found in syntenic regions of each genome and three (*hopM*, *avrE* and *hopAA*) are closely linked to TTSS structural genes, as noted [49]. A second class of TTE families (*hopX*, *hopAE*, *hopAF*, *hopR*, *hopAS*, *hopAB*, *hopQ*, *hopD*, *hopT*, *hopO*, *hopW*, *hopF*, *hopV*, *hopAZ*, *avrPto*) are predominantly absent from group II strains. They are located in a wide variety of genomic locations and can be very diverse in sequence, suggestive of extensive horizontal transfer (see below). Sequence differences among members of these families suggest that this class of TTE families may be under different evolutionary pressures relative to the core TTEs (Table S3, Figure S14).

**Figure 4:**
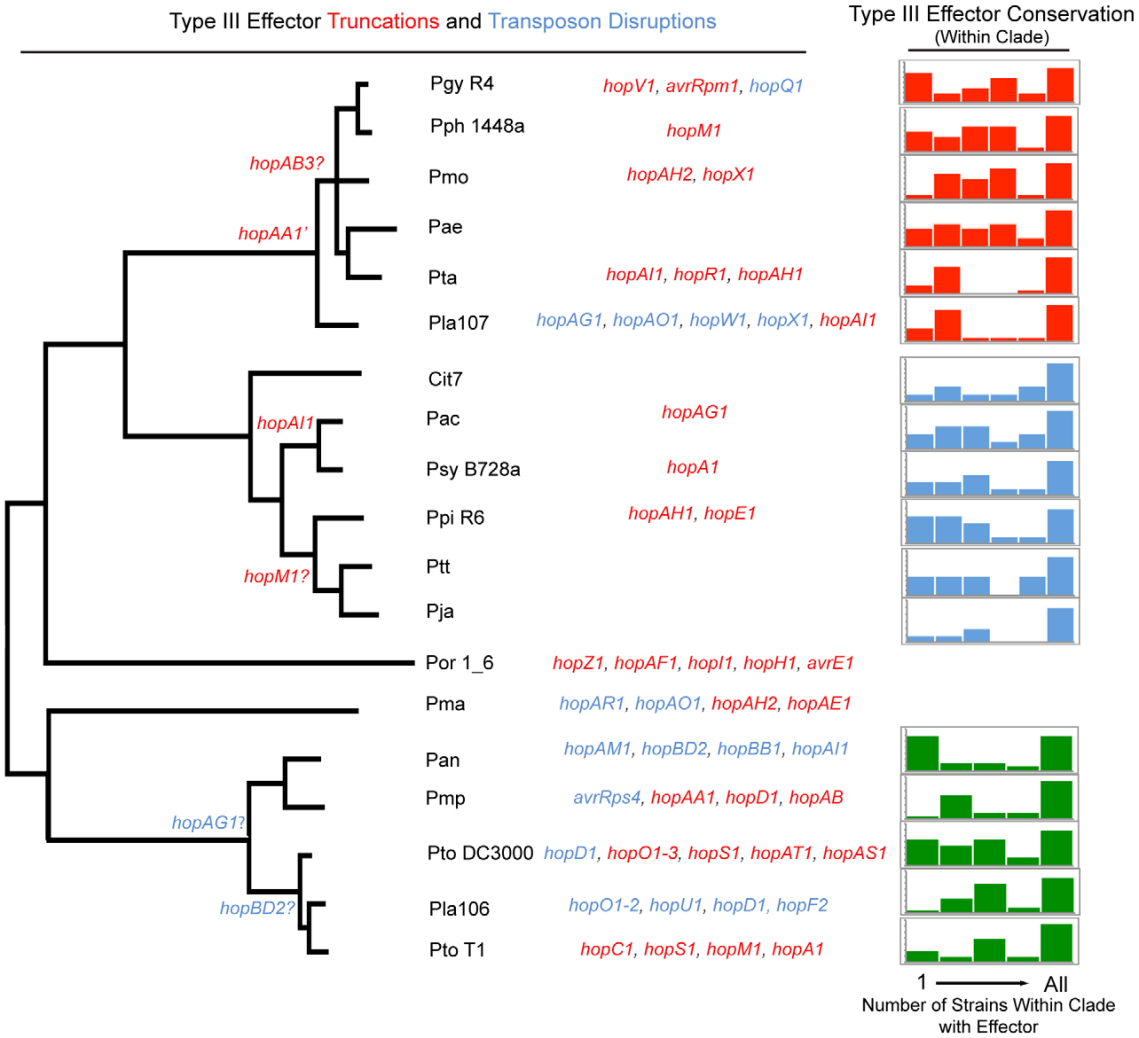
Phylogenetic conservation of disrupted and functional TTE proteins. Parsimony was used to determine at which phylogenetic nodes TTE disruptions occurred according to the phylogeny in Figure 1. The gene names of TTEs that are truncated are displayed in red, while those that are disrupted by insertion sequence elements are in blue. TTE disruption events that could not be phylogenetically placed, and presumably occurred only in one strain, are listed to the left of the phylogeny. Question marks next to the TTE name indicate that there is conflicting information concerning disruption or disruptions that could not be verified. We include a disruption of *hopF2* from *Pla* 106 because, even though the TTE sequence is complete, there is a transposon disruption in the *shcF* chaperone. At the far right, TTE conservation was determined for each genome within *P. syringae* groups where multiple strains were sequenced (groups I, II, III). The graph displays the percentage of each strain's TTE repertoire shared with other sequenced strains within an MLST group. The X-axis of this graph displays the number of potential strains within each MLST group that share particular TTEs with the genome of interest (max of 5 for group I and 6 for groups II and III). The percentage of singleton TTEs (found only within the strain of interest) is at the far left side of the graph, while the percentage of a strain's TTE repertoire conserved throughout the group is at the far right. The Y-axis represents the percentage of the TTE repertoire for each strain shared with other strains within the same MLST group and is scaled differently on each graph, however, the total area represented by each graph is 100% of the total effector repertoire for each strain.

We investigated the genome dynamics of TTE genes within the three most deeply sampled clades in order to characterize the evolution of TTE repertoires (Figure 4). For each strain, the majority of the TTE ORFs are present within other closely related strains from that clade. Moreover, most strains share almost all of their TTEs with at least one additional strain from within the same clade. This result is particularly striking for *Pta*, *Pla* 107, *Pmp*, *Pla* 106, *Ptt*, *Pja*, *Cit7*, *Pac*, and *Psy* B728a, which only have a small percentage of novel TTEs in relation to the rest of their clade (Figure 4, right). In contrast, a handful of isolates (*Pgy* R4, *Pph* 1448a, *Pae*, *Ppi* R6, *Pan*, *Pto* DC3000) have gained numerous TTE that are not present within any other related strains.

TTE truncations and transposon disruptions are common across the *P. syringae* phylogeny (Figure 4). However, in only two cases, a truncation of *hopAA1* and a transposon disruption of *hopAG1*, are these events shared by a majority of strains within a clade. Similarly, in only three other cases did events occur that were shared between multiple strains. Conversely, of the 46 total TTE gene truncations or transposon disruptions we identified, 41 appear at the tips of our phylogeny. Given the rarity of “older” truncations and disruptions, and given that many of these altered TTEs are found undisturbed in closely related genomes, we believe TTE loss is recent in most cases. This is consistent with ongoing dynamism in host range determination, whether across plant species or within a species, driven by host immune recognition. Additionally, there is a distinctive proliferation of IS element disruptions amongst the group I strains. The other clades appear to display higher rates of disruption by truncation (mostly via frameshift mutations) than IS elements, with the exception of *Pla* 107 which possesses a relatively high number of TTE with IS element disruptions. This trend potentially reflects differences in the activity levels of clade specific transposases.

### Identifying the Most Evolutionarily Dynamic Type III Effector Families

We investigated diversity for 35 TTE families (Table S3) present within a majority of strains (>12) by calculating measurements of pairwise amino acid diversity among all known alleles (Table S3). The most diverse TTE families are *hopW*, *hopZ*, *avrB*, *hopAO*, *hopT*, *hopAB* and *hopF* (Table S3). The diversity values for *hopW*, *hopT* and *hopAO* are somewhat misleading, however, as these families contain alleles of vastly different lengths. We built phylogenies from protein sequences of the remaining diverse TTEs (*hopAB*, *hopF* and *avrB*) (Figure S14) and note that similar analyses exist for *hopZ* [36]. The resulting phylogenies differ extensively from the phylogenies inferred from MLST sequences, hence we infer that these widely distributed TTE families are often lost, but can be regained by horizontal transfer of divergent alleles (Figure S14). This could imply that these TTE families play important roles in virulence across a broad range of host species, and are thus often re-incorporated into a strain's TTE suite. But the dynamism of these families also suggests that they may be evolutionarily costly on certain hosts (again likely through host immune recognition) and are therefore lost at higher rates than other TTE families. In support of this model, we note that host disease resistance genes exist that recognize specific members of each of these four TTE families, and that specific members of each of these TTE families can confer virulence on particular hosts [2], [50], [51].

### Phylogenetic Distribution of Non-TTSS Secreted Virulence Factors

We investigated the presence of pathways encoding the best understood *P. syringae* phytotoxins (coronatine, tabtoxin, syringolin, syringopeptin, syringomycin, phaseolotoxin), a gene (*avrD*) whose enzymatic product, syringolide, can cause a hypersensitive response on specific soybean genotypes [52], and genes involved in production or modification of the plant hormones ethylene and auxin (Figure 3). It should be noted, however, that allelic diversification within these pathways can lead to the production of slightly different toxins [53]. In most cases, pathways coding for toxins found together in *Psy* B728a (syringomycin, syringopeptin, syringolin) are found in the genomes of group II strains, with the exception of *Ppi* R6. In only one other case did a strain contain genes known to be involved in the production of multiple toxins (coronatine and tabtoxin in *Por*). Moreover, although all strains in all groups appear capable of producing the plant hormone auxin, only the group II strains and *Pph* 1448a lack a gene to modify auxin once it is made (*iaaL*). *avrD* is widespread throughout the phylogeny, although the functional significance of different *avrD* alleles remains unresolved [52]. Only *Pgy* R4 and *Ppi* R6 appear to be capable of ethylene production by known pathways. The relative wealth of phytotoxins and the reduced TTE suites of group II strains, compared to the other phylogenetic groups, suggest that the genetic basis of pathogenicity within this clade has diverged from the rest of the *P. syringae* species.

### Associations in the Distribution of Virulence Genes

We analyzed the relationships of virulence gene suites across strains by hierarchical clustering of strains with respect to the distributions of individual virulence genes and pathways (TTE, phytotoxin pathways, plant hormone mimics) (Figure S13). Although we hoped to uncover novel associations between virulence genes, small sample sizes for numerous virulence genes provide little resolution to identify correlations that are independent of phylogeny (i.e. *hopN*, *hopS*, *hopY*) or known proximity on the chromosome (i.e. *hopO* and *hopT*). However, clustering of strains by their virulence gene repertories does highlight some interesting trends. Despite phylogenetic assignment to group I, both *Pan* and *Pmp* diverge from *Pto* T1*/Pto* DC3000*/Pla* 106 in their virulence gene repertoires. Such patterns reflect the classification of these strains within different genomospecies [20]. Indeed, *Pan* clusters more closely with group III strains, likely due to the presence of scaffolds and TTE's related to those found on the large virulence plasmid of *Pph* 1448a, as well as the pathway for the production of phaseolotoxin. This could signal underlying similarity in the virulence strategies of *P. syringae* pathogens of beans and kiwi. Likewise, two group III strains (*Pta* and *Pla* 107) cluster with group II strains based on virulence gene profiles, suggesting that there is a fundamental difference in virulence gene repertoire for these two strains compared to their group III relatives. Lastly, hierarchical clustering clearly demonstrates divergence in virulence genes suites between *Ppi* R6 and other group II strains.

### Identifying Evolutionarily Dynamic TTEs Based on DNA Sequence Diversity within Clades

**Figure 5:**
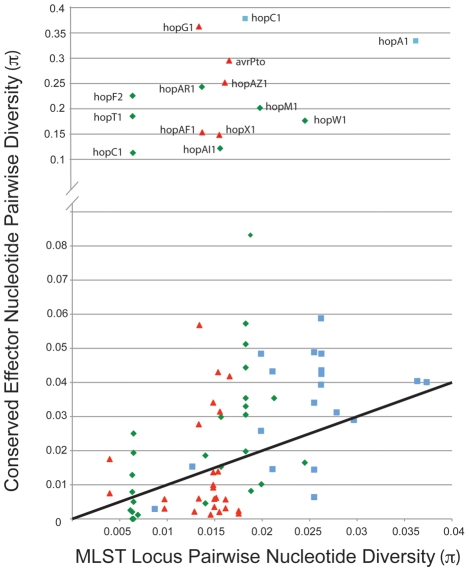
A minority of shared TTE alleles display elevated levels of nucleotide divergence. Pairwise nucleotide diversity values (π) were calculated between TTE genes shared across multiple strains within each *P. syringae* groups, color coded by phylogenetic group as in Figure 1, and compared to (π) values for housekeeping gene fragments from these same strains. The solid line indicates a 1∶1 ratio of π values between housekeeping genes and shared TTE. Only effector families with high π values from within phylogenetic groups are labeled.

We compared values for π (pairwise nucleotide diversity) for shared TTE subfamilies within the three clades of *P. syringae* for which there are genome sequences from multiple strains (Figure 5). As a baseline, we compared this value to the π values for the same groups of strains calculated from the concatenated MLST loci used to construct the overall phylogeny in Hwang et al. [16]. This metric requires accurate placement of TTEs into subfamilies ([48]; Materials and Methods). Thus, there is an upper limit to π values for the shared TTEs because extremely divergent alleles will be placed into different subfamilies. A majority of the π values for the TTE subfamilies match, or are slightly higher than, values for the housekeeping genes, consistent with vertical inheritance or low levels of diversifying selection. However, there are numerous instances where diversity within a TTE subfamily far exceeds the diversity values for the housekeeping genes within the same comparison. Future work will show whether horizontal transfer or mutation has enabled functional diversification of these protein families.

### The Evolutionary Dynamics of *hopM1* within Group I Strains

**Figure 6:**
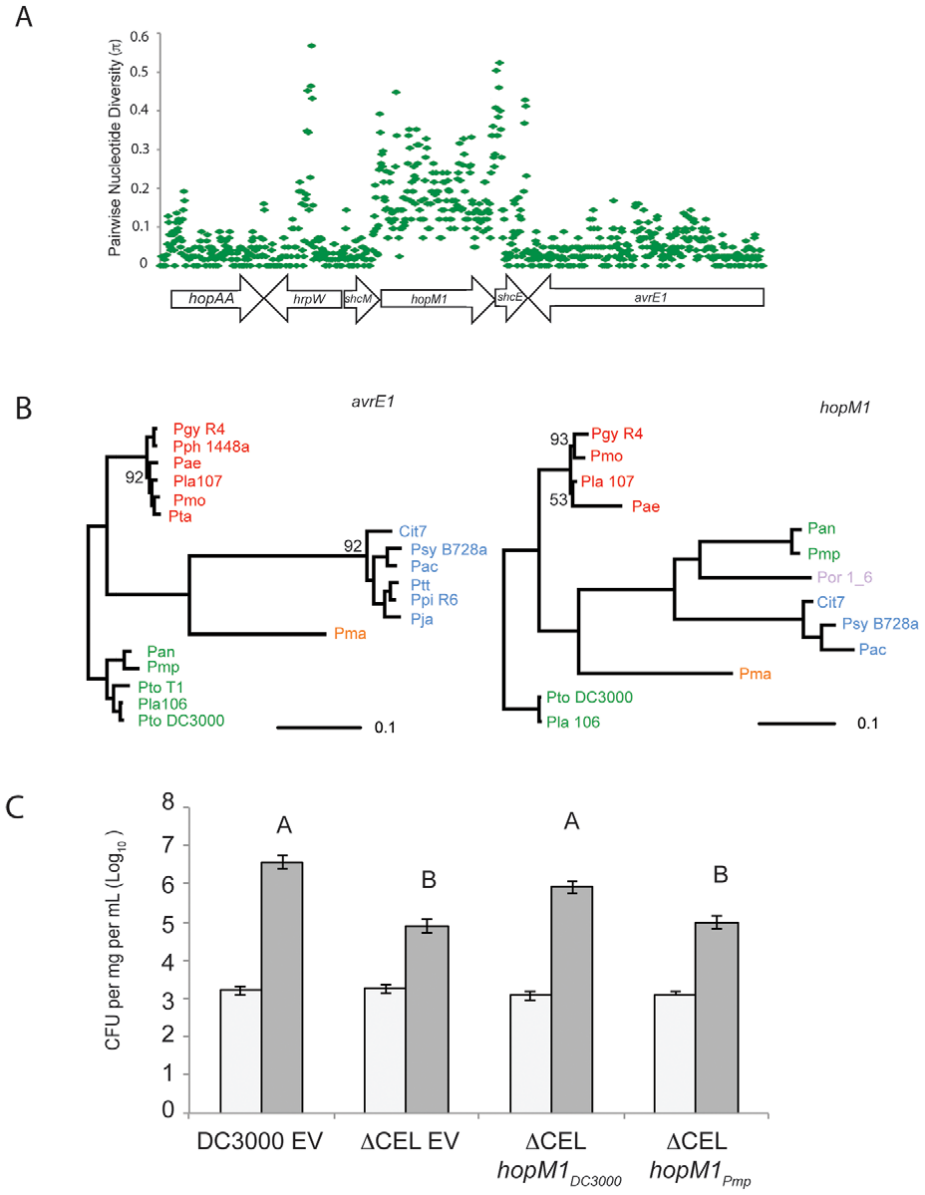
Recombination of *hopM1* in group I strains leads to functional divergence. (**A**) A genomic region including *hopM1* was aligned for all group I *P. syringae* strains with draft genome sequences and pairwise nucleotide diversity values (π) were calculated in 25 bp sliding windows. (**B**) Phylogenies were constructed using Bayesian methods for both *avrE1* and *hopM1* for all publicly available alleles. Posterior probabilities are shown if support for nodes is <0.99. Color-coding of strain names represents phylogenetic group designation as described in Figure 1. (**C**) *schM/hopM1* from *Pmp* is unable to complement the virulence defect of *Pto* DC3000 ΔCEL in dip assays on Arabidopsis. Bars indicate mean growth at zero and four days after inoculation. Error bars are 2× standard error. Different letters indicate statistically significant differences (ANOVA, Tukey's HSD).

Our sequence diversity analysis showed that *hopM1* has experienced unusual evolutionary dynamics (Figure 5), especially within the group I strains. We aligned the largest contiguous genomic region (bordered by scaffold breaks) including *hopM1* for all group I strains that contain a full-length *hopM1* allele. We computed π values for the nucleotide sequence of this region for these strains. A small fraction of this genomic region, which includes *hopM1* as well as fragments of the TTSS helper protein *hrpW* and the TTE chaperone *shcE*, displays inflated π values relative to the bordering regions (Figure 6A). Therefore, the observed inflation of nucleotide diversity for *hopM1* (Figure 5) is localized. The phylogenies of both TTSS linked TTEs *avrE1* (Figure 6B) and *hopAA1* (data not shown) match those created from MLST loci. However, the *hopM1* phylogeny shows that a recombination event involving the *hopM1* locus splits group I strains into two divergent groups (*Pmp*/*Pan* or *Pto DC3000*/*Pla* 106/*Pto* T1) (Figure 6B, right, in green). This result underscores how localized homologous recombination of existing sequences can drive diversification and adaptation of *P. syringae* TTE repertoires [54].

### Allelic Variants of *hopM1* are Functionally Diverged

We tested the virulence function of both of the diverged group I *hopM1* variants, from *Pto* DC3000 and from *Pmp* using a previously published assay [55]. Briefly, a strain carrying a deletion of the *C*onserved *E*ffector *L*ocus (CEL) that eliminates *hopM1* and *avrE1* from *Pto* DC3000 displays attenuated disease symptoms and less growth on Arabidopsis. *avrE1* and *hopM1* are likely redundant for this virulence function [14], [56], [57]. We found that *hopM1_Pmp_*, expressed from a constitutive promoter, did not complement the virulence defect of *Pto* DC3000 ΔCEL (Figure 6C), even though this effector is translocated (Dataset S9). Therefore, allelic variants of *hopM1* display functional divergence for virulence on tomato.

### A Natural Allelic Series of AvrPto Orthologs Naively Recapitulates Identification of the GINP Loop as a Region Critical for AvrPto/Pto Avirulence

**Figure 7:**
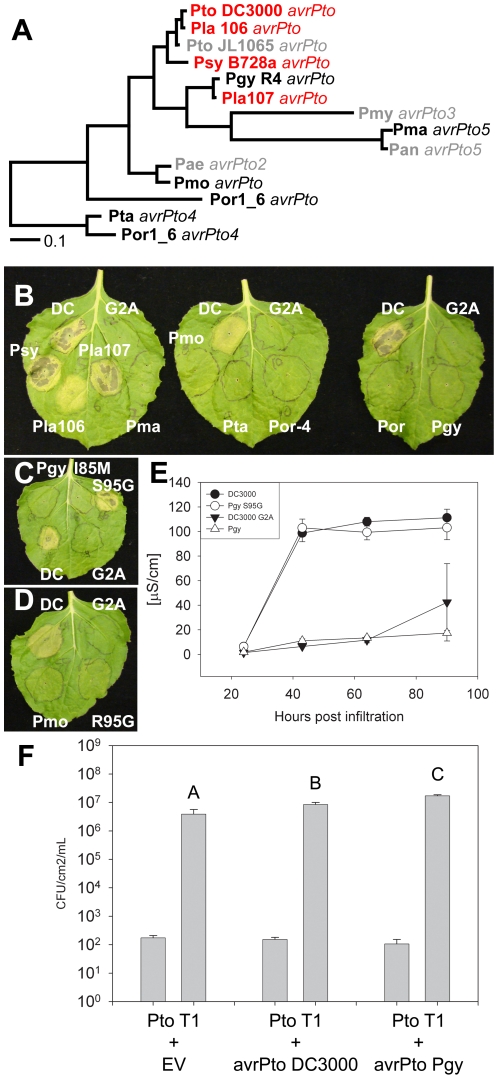
Phylogenetic analysis of the AvrPto superfamily reveals a residue required for avirulence function. (**A**) Bayesian phylogeny for the AvrPto superfamily. Orthologs in red are recognized by Pto, orthologs in black are unrecognized, while orthologs in gray are untested. (**B**) Agrobacterium/*N. benthamiana* transient assay. Indicated AvrPto orthologs were co-expressed with Pto and symptoms are assessed at 4 days post innoculation. The *Pto* DC3000 AvrPto G2A mutant is a mislocalized negative control. (**C**) Mutation of S95G restores activity to the unrecognized ortholog AvrPto*_Pgy_*_R4_. Mutation of M85I does not result in recognition of AvrPto*_Pgy_*_R4_. (**D**) Mutation of R95G does not restore recognition of the AvrPto*_Pmo_* ortholog. (**E**) Ion leakage assay of Agrobacterium/*N. Benthamiana* transient inoculations. Error bars are one standard deviation. (**F**) Expression of either AvrPto*_Pto_*_ DC3000_ or AvrPto*_Pgy_*_R4_ is sufficient to increase the virulence of *Pto* T1 on tomato plants incapable of recognizing AvrPto (tomato cultivar 76R *prf3*). Bars indicate growth at zero and four days after dip inoculation with 10^5^ CFU/mL bacteria. Error bars are 2× standard error.

To show the utility of deep phylogenetic sequencing, we asked if a diverse collection of orthologs could be used to predict functional information about a TTE protein. AvrPto is a widely distributed and well-characterized TTE that interacts with a tomato host cellular target, the protein kinase Pto, in a well-defined manner that results in disease resistance mediated by Pto and the NB-LRR immune receptor Prf [58], [59]. AvrPto also confers added virulence to *P. syringae* strains that lack it when assayed on *pto* or *prf* genotypes of tomato [60]. We assayed 10 AvrPto orthologs (Figure S16A, Figure 7A) for their ability to trigger Pto dependent HR using a standard assay in *N. benthamiana* [61]. As expected, AvrPto from *Pto* DC3000 and closely-related orthologs triggered Pto-dependent HR. More distant orthologs did not (Figure 7B). A negative control, AvrPto_DC3000_ with a G2A mutation, previously reported to be mislocalized [62], failed to elicit HR in the presence of Pto. Expression of AvrPto orthologs and Pto was verified by Western blotting (Figure S16B).

One allele that was not recognized by Pto, AvrPto*_Pgy_*_R4_, differs in only 2 residues from the recognized allele AvrPto*_Lac_*_107_ (Figure S16A). We used phylogeny-directed mutagenesis to create AvrPto*_Pgy_*_R4_ I85M and AvrPto*_Pgy_*_R4_ S95G, recreating the conserved sequence of the recognized AvrPto orthologs in each case. We tested the ability of these alleles to be recognized by Pto. AvrPto*_Pgy_*_R4_ I85M was not recognized, while AvrPto*_Pgy_*_R4_ S95G triggered Pto-dependent cell death (Figure 7C). In a second assay for HR, we found that, while AvrPto*_Pgy_*_R4_ was inactive, AvrPto*_Pgy_*_R4_ S95G induced ion leakage to levels indistinguishable from AvrPto*_Pto_*_DC3000_ (Figure 7E). Glycine 95 is common to all the active AvrPto alleles and lies within the GINP loop, a region required for the AvrPto-Pto physical interaction and hence avirulence function [58], [63]–[65]; Figure S16C). Recently, Glycine 95 has been shown to be required for recognition of AvrPto by Pto [66]. Both Serine 94 and Isoleucine 96 are required for avirulence [64]. Isoleucine 96 has been previously shown to tolerate mutation to valine (but not alanine) [64]. Accordingly, while both AvrPto*_Lac_*_107_ and AvrPto*_Pgy_*_R4_ S95G contain valine at position 96, they both trigger avirulence. A more distantly related, non-recognized allele, AvrPto*_Pmo_* also contains a non-consensus arginine residue at position 95. Mutation of AvrPto*_Pmo_* R95G did not restore recognition (Figure 7D). Thus, other AvrPto*_Pmo_*-specific polymorphisms contribute to loss of recognition. The non-recognized ortholog AvrPto*_Pgy_*_R4_ retains its virulence function on tomato leaves that lack Pto function (Figure 7F), suggesting that the two amino acid differences that distinguish it from AvrPto alleles that are recognized are dispensable for virulence. A similar separation of AvrPto avirulence and virulence functions has been previously reported for missense mutations of the canonical AvrPto allele from *Pto* JL1065 [64]. The virulence effect of AvrPto*_Pgy_*_R4_ was consistently greater than that of AvrPto*_Pto_*_C3000_ (Figure 7F). This relative difference could be due to either residual avirulence of the *Pto* DC3000 ortholog dependent on glycine 95, or to uncharacterized residues polymorphic between AvrPto*_Pgy_*_R4/*Lac*107_ and AvrPto*_Pto_*_C3000_.

## Discussion

Bacterial genomes are dynamic. Large-scale changes occur rapidly and differentiate even closely related isolates within the same species. *P. syringae*, an important pathogen of many plant species, is a diverse assemblage of strains isolated from different host plants as well as from the environment. To reveal the evolutionary history of pathogenesis within this species, we catalogued the virulence gene repertoires for 19 isolates using genome sequencing coupled with a nearly saturating screen for novel TTE families for a subset of the strains. These phylogenetically diverse genome sequences provide a comprehensive comparative tool to investigate pathogenicity and virulence across plant hosts and a means to gain insight into host range and adaptation of this important phytopathogen.

### Genome Structure and Diversity

Although individual isolates of *P. syringae* contain upwards of 6000 genes, only 3397 are shared amongst all 19 sequenced strains (Figure 2A). While estimates of core genomes typically decrease with further sampling from diverse isolates, we do not expect the *P. syringae* core to significantly decrease, given the slope of the data in Figure 2 and our sampling of much of the known phylogenetic diversity of pathovars. For comparison, we identified species-specific core genomes, using the same methods, from multiple sequenced genomes of both *P. fluorescens* (a plant-associated microbe) and *P. putida* (a soil bacterium) as well as the subsets of genes shared between different combinations of these species (Figures 3C, S7, S8). Both the unique portion of the *P. syringae* core and the core genes shared between *P. syringae* and *P. fluorescens*, are enriched for proteins involved in protein localization and transport (comparing Figures S7, S8, S9), highlighting the potential role for such processes in adaptation to plant hosts. Surprisingly, the core genomes for both *P. putida* and *P. fluorescens* are larger than that for *P. syringae* (Figure 2C), which could reflect differences in evolutionary pressures for the pathogenic strains or the smaller number of sequenced genomes for *P. putida* and *P. fluorescens* (the number of core genes could drop substantially with further sampling). *P. syringae* group I strains share ∼500 more genes than either of the other two well sampled groups (Figure 2D). The majority of these clade-specific genes encode proteins of unknown function (Figure S10).

The 19 *P. syringae* strains define a larger core genome than do 20 strains of *Escherichia coli* (3397 vs. 1976) but a substantially smaller pan-genome (12,749 vs. 17,838) [67]. These numbers are surprising given the larger overall genome for pseudomonads in general (average of ∼6000 genes compared to ∼4700 for *E. coli*). Therefore, even though pseudomonads are ubiquitous across many environments, it is possible that *E. coli* fills more diverse ecological niches, requiring lower numbers of shared genes but correspondingly higher numbers of unique pathways. However, we have only sequenced isolates from crops and the size of the core genome may be reduced when sampling from more diverse environments.

Plasmids, which contain many pathogenicity genes and have the potential for horizontal transfer across strains, are known determinants of virulence evolution within *P. syringae* [45]. However, plasmids are often filled with repetitive regions that make assembly from short-read sequencing data difficult. We have attempted to identify plasmid regions using a combination of presence of conserved plasmid genes as well as sequencing coverage levels. However, we ultimately found that it was difficult to truly identify presence of plasmids using assembly information alone. The best strategy for sequencing of these difficult regions may still be to isolate individual plasmids and sequence them separately.

Not surprisingly, 13 of the 15 pathovars show evidence of plasmids that are of the same approximate size and genomic composition as previously identified or sequenced *P. syringae* plasmids (Table S1). They contain TTE loci and therefore likely contribute to virulence, as noted previously [45]. Moreover, the backbone and many virulence genes found on the large plasmid of *Pph* 1448a are present within *Pgy* R4, *Pmo*, and *Pan* and this could reflect a larger role for this plasmid as a virulence factor in multiple host species than previously recognized. Indeed, the virulence gene suite of *Pan* is more similar to these group III strains than other more closely related strains (Figure S13).

We also found a recently acquired 1 Mb megaplasmid in the cucumber isolate *Pla* 107, and in a closely related strain also isolated from diseased cucumber. This megaplasmid is absent from two other cucumber isolates (Figure S12) and appears to possess the same copy number as the chromosome (Figure S11). This finding was both unexpected and unprecedented, as previously identified megaplasmids are typically conserved within all isolates of a species [68]. Megaplasmids can facilitate dramatic ecological shifts within bacteria [68], but we have not been able to predict phenotypic changes from pathways present on pMPPla107. Additionally, this megaplasmid contains a complete type IV secretion system (TFSS) most closely related to the Dot/ICM system from *L. pneumophila*. It is unknown whether this TFSS is used strictly for self-transmission and conjugation or if it actively secretes effector proteins. Although we did not find evidence for known type IV effectors on the megaplasmid, presence of this TFSS could represent a completely new contributor to virulence within *P. syringae*.

### Identification and Distribution of Pathogenicity Factors

The primary determinants of virulence in *P. syringae* are TTEs and phytotoxins. Combining a high-throughput promoter trap screen with draft genome sequences for a subset of strains, we identified eight new TTE families (Table 2, Figure 3). In sum there are now 58 (not including *avrD*) TTE families across these 19 strains [48]. As we identified only nine new TTE families by screening these phylogenetically diverse strains, we believe that we have nearly saturated the discovery of TTE families found within *P. syringae*. Furthermore, additional candidate loci identified in our functional screen as HrpL-regulated were not translocated (Dataset S9). Each of these loci with non-translocated proteins possesses a functional *hrp*-box, linking gene expression to a known pathogenicity regulon, and therefore implicating these genes as virulence factors. Moreover, some of these loci have not been identified in previously sequenced genomes or by previous screens. We are confident that we have an exhaustive list of potential effectors for most of the sequenced strains. However, there is still likely to be substantial undiscovered diversity in the HrpL regulon across *P. syringae*.

Comparisons of evolutionary rates for TTE families shared throughout the *P. syringae* phylogeny could reveal specific TTEs important for virulence on a particular host. Yet, the virulence activity of any TTE can drive strong selection against its presence in a pathovar if that activity leads to recognition by a plant immune receptor. To capture this dynamic, we analyzed two classes of TTEs present within the 19 genomes. First, TTE effector families with wide distribution and very little divergence likely perform conserved virulence functions in a range of plants, and may additionally be evolutionarily ‘unrecognized’ across a wide range of plant hosts. Surprisingly, there are only five core TTE genes present in all pathogenic strains (Figure 3), and at least 4 of these have known virulence functions in *A. thaliana* [14], [55], [69], [70]. By virtue of their positional orthology in each genome, these few TTE potentially provide basic virulence functions.

Second, TTE genes found at different genomic locations in many strains, encoding proteins that are highly divergent (Table S3), could be under intense host selection driving diversification. This may mean that these TTEs have great potential to limit growth or help a pathovar expand across hosts. These widely distributed yet diverse TTE families could represent a class of virulence genes specialized to target rapidly evolving plant genes or pathways. They could, therefore, be most responsible for large-scale differences and limitations in host range. Interestingly, two of these, *avrPto* and *hopAB* are known to interact with common, and highly diverged, host PRR kinase domains to suppress host defense [71]–[74]. The rapid evolution within these TTE genes suggests that these TTEs are also widely recognized by the host immune system, leading to rapid loss, replacement, gain and, potentially, diversification. The most divergent TTE families are also experiencing high levels of horizontal gene transfer since their evolutionary histories do not mirror the phylogenies of the respective housekeeping genes (Figure S14).

Broadly, group II strains (including the completely sequenced isolate *Psy* B728a) contain fewer TTEs on average than the other clades (Figure 3). We hypothesize that strains within group II rely more heavily on non-TTSS based virulence factors for virulence as almost all members of this group share two of three known phytotoxin pathways. Indeed, the one strain from this group with the most TTEs (*Ppi* R6) is the only strain lacking genes for these pathways from this group. Furthermore, although all strains contain genes for the production of the plant hormone auxin, which can be an important virulence factor, only group II strains and the bean pathogen *Pph* 1448a lack a gene for auxin modification. Taken together, strains in this clade have apparently shifted their mechanisms of pathogenesis through TTE loss coupled with acquisition of phytotoxins by an ancestor of group II. In support of this hypothesis, we note a recent report where syringolin A modifies stomatal function in a manner that is phenotypically similar to, but mechanistically independent of, coronatine [75].

The smaller TTE repertoire of group II strains is not a sampling artifact. First, the gold standard genome of strain *Psy* B728a has been searched for the presence of TTEs by both experimental and bioinformatic methods [25]. Only 16 TTEs are found within this strain, still significantly less than most of the strains from the other phylogenetic clades. Secondly, we thoroughly sampled *Psy* B728a and other strains from this group (*Pac*, *Ppi* R6, *Ptt*, *Pat*) in our screen for novel TTE (Table S2). Thirdly, strains within group IIC have lost the canonical type III secretion system and most associated TTEs but maintain phytotoxin pathways [18]. It is unlikely that the progenitor of this group lacked the canonical TTSS, because structural genes of the TTSS (data not shown) as well as linked TTEs (*avrE1*, *hopAA1*, *hopM1*) appear to have been vertically inherited within this group, including strain Cit7 which diverges earlier than strains with an atypical TTSS (Figure 6B).

There is no pattern in the distribution of the remaining phytotoxin pathways in these sequenced strains. Excluding the group II clade and *Por* 1_6, remaining strains do not harbor multiple pathways for the production of known phytotoxins (Figure 3). As previously reported, only *Pph* 1448a and *Pan* share the genes involved in phaseolotoxin production. These pathovars also share many TTE loci commonly found in group III (Figure S13). Since *Pan* appears to have recently acquired these TTE genes (many of which may be present on a plasmid that resembles the large virulence plasmid of *Pph* 1448a), it is possible that phaseolotoxin and these shared TTE families target complementary host defense functions.

Virulence genes that modify plant hormonal pathways are also evolutionarily dynamic (Figure 3). Both *Pgy* R4 and *Ppi* R6 have recently acquired a gene involved in ethylene production. Ethylene production is thought to suppress host responses to biotrophic pathogens [76]. *P. syringae* strains are biotrophic, at least early in their life cycle on plants. The importance of coronatine, a structural mimic of the plant hormone jasmonic acid, as a virulence factor during *Pto* DC3000 infection of Arabidopsis has been noted [34]. Our data suggest that coronatine biosynthesis is a recent import into the *Pto* DC3000 genome and its HrpL regulon. *Pto* DC3000 mutants deficient in the production of coronatine are impaired during invasion via stomata, but are capable of causing disease if delivered directly into the host tissue [34]. Thus, absence of the coronatine pathway may partially explain why the *Pla* 106 and the tomato pathogen, *Pto* T1, are not virulent in Arabidopsis.

### Host Range Evolution

Host range is notoriously difficult to define because strains could be pathogenic on unrelated, unknown, and invariably untested, host plant species. Additionally, there may be quantitative differences in pathogen growth on a given host species, even among strains grouped as non-pathogens. Furthermore, basic evolutionary questions such as the plasticity of host range and the number of steps involved in adaptation to a new host remain unanswered [4]. However, as the HrpL regulon is important in structuring host range and promoting virulence [4], [8], [29], understanding the evolution of TTE repertoires can reveal the *potential* for host range evolution across the *P. syringae* phylogeny.

Across three well-sampled clades of the phylogeny, the majority of the TTE suites for each strain are shared between at least two other strains within each group (Figure 4). In only a few cases do singleton TTEs make up a significant part of the total TTE repertoire of each strain. Although only a small number of strains have a high proportion of singleton TTEs, singletons may indicate recent shifts in the host ranges of these strains mediated by TTE gain. They are, therefore, important targets of future research into virulence mechanisms. Other isolates form well-defined groups according to conservation of TTEs (Figure 4, S12). These strains potentially utilize similar virulence strategies during infection, which limits the potential for host shifts among their particular host plants. For instance, *Pla* 106 was isolated from diseased cucumbers but shares much of its TTE suite with two tomato pathogens. Thus, host shifts might be more likely between tomato and cucumber because these strains carry similar suites of TTEs. Furthermore, as noted above, host range shifts or pathogenicity across bean and kiwi plants may be correlated due to the recent acquisition by *Pan* of a plasmid likely containing many virulence genes found in *Pph* 1448a as well as the pathway for phaseolotoxin production.

In striking contrast to the conservation patterns of functional TTE is the diversity of TTE inactivation due to truncations and transposon insertions (Figure 4). Because any given TTE can trigger a specific immune response during infection, inactivation of TTEs (*i.e.* removing the trigger) may play an important role in broadening and maintaining host range [25], [77]. Inactivated and truncated TTEs are more frequently found at the tips of the phylogeny than at internal nodes. Two non-mutually exclusive possibilities can explain this trend; the majority of TTE disruptions occurred recently or inactivated TTE are rapidly purged from the genome [78]. If the rate of TTE truncation and pseudogenization is truly higher at the tips of the phylogeny, then the lack of recognized TTE may be more important for recent changes in host range than the presence of functional TTE.

Recent studies using *Xanthomonas* suggested that isolates convergently evolved to infect the same hosts have acquired very similar sets of TTE [79]. To test this idea in *P. syringae*, we compared the TTE repertoires of *Pla* 106 and *Pla* 107, two distantly related strains from designated as the same pathovar (Figure S15). Five shared TTE families are common to these two strains that could act as general cucumber virulence factors. Three of these appear to be fairly recent acquisitions within both of these strains, in that they are only present within a limited number of other strains within the clade (*hopE1*, *hopA1*, *hopBD1*). Furthermore, *hopAG1* is a TTE that has been convergently lost in each of these strains, suggesting that HopAG1 is recognized by cucumber. We tested for recognition of the *Psy* B728a *hopAG1* allele in *Pla* 107 during growth *in planta*, but found no effect (Figure S15). Although generalizations of the role of *hopAG1* in limiting host range on cucumber should include tests of multiple alleles on multiple cultivars of cucumber, these results suggest that *hopAG1* does not play a broad role in limiting host range for pv. *lachrymans* and such gene loss may just be coincident pseudogenization of unnecessary proteins.

### Type III Effector Function and Evolution

Host range could also be modified by diversification of shared TTE [36], [80]. Our draft genome data enable the identification of evolutionary signatures of diversification across shared alleles. Although pairwise diversity is slightly higher than the housekeeping loci for most TTE subfamilies, a handful of shared alleles display elevated levels of divergence, suggesting dramatic changes in specificity or function of TTE families.

In the *hopM1* subfamily, allelic diversification may contribute to host range. This is consistent with the positional conservation of *hopM1* across strains, its linkage to the TTSS-encoding pathogenicity island, and its defined virulence function in *Pto* DC3000. A fragment of HopM1 interacts with the *A. thaliana* protein AtMIN7, an ARF-GEF protein likely to be involved in vesicle transport and potentially in secretion of anti-microbial products [55]. As shown in Figure 6, it is striking that this TTE has undergone a clean gene conversion event while divergent alleles of other shared TTE are horizontally transferred to different places within the genome. While the *hopM1* allele from *Pto* DC3000 complements virulence deficiencies of the *Pto* DC3000 ΔCEL mutant, the divergent *hopM1* allele from *Pmp* does not (Figure 6C). Therefore, sequence divergence of *hopM1* within the group I strains leads to functional divergence during Arabidopsis infection. These diverse alleles could target different host proteins, have host-dependent specificities of interaction, for example with diverged AtMIN7 orthologs, or have functionally co-evolved with other virulence-related genes in these strains. Interestingly, *avrE1* from the *Pmp*/*Pan* clade appears to be vertically inherited from the ancestor of the group I strains suggesting that it would complement the *Pto* DC3000 ΔCEL virulence deficiencies (Figure 6B). In this case, the functions of the *Pmp* alleles of *hopM1* and *avrE1* are not likely to be redundant, in contrast to the finding that either allele from *Pto* DC3000 can complement *Pto* DC3000 ΔCEL [14], [55], [56]. Given the high levels of divergence across the *hopM1* sub-family, it is difficult to pinpoint causal amino acid changes for the virulence defects. As this example illustrates, evolutionary divergence among shared TTE could structure changes in host range and pathogenicity. Our unbiased measurements of diversity are a first step towards identifying TTE families with interesting evolutionary signatures.

Our deep phylogenetic sequencing generated a large collection of orthologs. These orthologs are a natural allelic series. As a test case, we used AvrPto to see if its natural diversity could uncover important functional information. AvrPto has been extensively studied, and its physical interaction with Pto has been characterized by both mutagenesis and co-crystallization [58], [60], [64]. We found that the orthologs of AvrPto most closely related to AvrPto*_Pto_*_DC3000_ were able to trigger a Pto-dependent HR. The most informative orthologs were AvrPto*_Pla_*_107_ and AvrPto*_Pgy_*_R4_. AvrPto*_Pla_*_107_ triggered a Pto-dependent HR, while AvrPto*_Pgy_*_R4_ did not. Both of these alleles are divergent from the *Pto* DC3000, *Psy* B728a and *Pla* 106 group, but relative to each other they are only polymorphic at 2 residues (positions 85 and 95); these are conserved in all AvrPto proteins that cause Pto-dependent HR. Both residues lie in or near the previously characterized AvrPto-Pto interaction surface. By individually mutating these two residues back to the consensus residue, we demonstrated that G95 is required for recognition, while M85 is not. G95 is in the GINP loop critical for AvrPto-Pto interaction. Thus, isolation of a natural allelic series allowed us to locate the binding surface on a TTE required for recognition by a host protein. This approach is generalizable to uncharacterized TTEs, given the identification of assayable host response.

The hypothesis that the G95 residue of an ancestor of the Pgy R4 ortholog has evolved to avoid recognition by a Pto/Prf-like system is consistent with the AvrPto phylogeny. Our virulence assay on tomato indicates that, consistent with previous studies, AvrPto is capable of mutation away from avirulence, while retaining virulence. The Pgy R4 ortholog is a striking evolutionary confirmation of the generation of avirulence-compromised, but virulence-competent mutants of AvrPto [64]. These data suggest that, at least in the case of AvrPto/Pto, *P. syringae* may be capable of quickly evolving at the level of a single amino acid to evade host *R-gene* recognition.

### Conclusions

*P. syringae*, a phytopathogen responsible for crop loss throughout the world, has evolved to live in a diversity of environments and infect a broad range of host plants. Although the evolutionary basis of host range determination is unknown for many pathogen systems, TTE and phytotoxins are thought to be the primary contributors within *P. syringae*. Here we uncover the evolutionary conservation of these virulence determinants across diverse strains of *P. syringae* using cost-efficient genome sequencing coupled with screening a subset of these strains for the presence of novel TTE proteins. We only found a small core of five TTEs, one of which is often disrupted, that were conserved across strains. We show that although TTE and phytotoxin repertoires change rapidly, overarching trends emerge for both TTE and phytotoxin content – such as the trade off between complexity of TTE content and the presence of phytotoxin pathways. These evolutionary trends are only apparent in the context of broad phylogenetic sampling of genome sequences. Furthermore, complete genome sequencing of diverse strains enables more refined investigation of shared virulence genes and also provides a framework to inform and identify novel structure-function relationships.

## Materials and Methods

### Strains

We chose strains for sequencing to maximize the sampling of genetic diversity and the variety of hosts of isolation across the phylogeny of *P. syringae* (Strain names and pathovar designations can be found in Table 1). It should be noted that *Pma* was originally misidentified as pathovar *maculicola* and actually should be classified as *P. cannabina* pv. *alisalensis*, but we maintain *Pma* nomenclature for consistency with previous reports [81]. It should also be noted that *Pae* is also known as strain NCPPB3681 [40]. Most surveyed strains have been categorized [3], [16]. Our characterizations of host range throughout the paper are largely inferred from pathovar designation and previously published results, and we did not extensively characterize host range for any strain. We minimized the amount of laboratory passage before sequencing (although we are unsure of exactly how long each strain has been passaged under laboratory conditions since isolation, and some of the sequenced strains are rifampicin resistant derivatives). Genomic DNA for construction of all sequencing libraries was from a single colony of each strain that was picked and grown overnight in 250 mL of King's B media shaking at 28°C. Genomic DNA was isolated using a CTAB genomic protocol.

### Sequencing, Assembly, and Annotation

Genome sequencing for all strains consisted of a minimum of 1 lane of unpaired 36 bp Illumina reads, in addition to ¼ plate of 454 Flex reads. For six of the strains (*Pgy* R4, *Pma*, *Pja*, *Pan*, *Ppi* R6, *Pla* 106) this ¼ plate of 454 was supplemented with an additional ¼ plate of 454 paired ends from a separate library. Paired ends for the remaining strains (*Pac*, *Pae*, *Ptt*, *Pmp*, *Pta*, *Cit7*, *Pla* 107, *Pmo*) were created using the Roche 454 Long Paired end protocol, and were thus part of the initial ¼ plate of 454. For *Pla* 107, additional Illumina runs (1 lane of each at 48 bp, 56 bp, 72 bp) were used to fill in scaffolds. Genome sequences of *Pta* and *Pma* were supplemented with genomic data from the same strains publically available from Genbank (*Pta*: NZ_ACHU00000000, *Pma*: NC_005922; NC_005921; NC_005920; NC_005919; NC_t005918) [30], [82]. A draft genome was also recently published for *Pae* [40]. All analyses for this strain were performed using only our own assembly, but the final assembly of this strain in GenBank includes genomic sequence from the other published sequence of the same isolate (NZ_ACXS00000000). A draft genome sequence was also recently published [38] for *Pgy* R4 (AEGH00000000), too late to be included in our assembly or analysis. Publically available genomic sequences for all assemblies can be considered at least high quality drafts [83].

The genomes of each strain were assembled using a modified version of the pipeline described in Reinhardt et al. 2009 [41]. All genomes were subject to all steps of the pipeline downstream of, and including, Newbler assembly. In some cases (*Pgy* R4, *Ppi* R6, *Pja*, *Cit7*, *Ptt*) VCAKE was used to build initial contigs from Illumina reads, while in other cases (*Pma*, *Pan*, *Pla* 106, *Pla* 107, *Pmo*, *Pmp*, *Pta*, *Pae*, *Pac*) EDENA was used to build these initial contigs [84]. We found that there was no large-scale difference in size or quality of contigs built with VCAKE or EDENA. Annotation was carried out by submitting all contigs and scaffolds for each of the draft genomes to the NCBI PGAAP pipeline (http://www.ncbi.nlm.nih.gov/genomes/static/Pipeline.html). NCBI accession numbers for each genome sequence are in Table 1 and protein sequences for all called ORFs as well as those fixed by Phylo-gene-boost consolidation (see below) are listed in Dataset S1, S2, S3, S4. Mauve alignments of draft genomes to complete genomes within each MLST group are presented for group I (Figure S6), group II (Figure S5), and group III (Figure S4).

### Phylogenetics

Fragments of nucleotide sequences for 7 genes previously used for MLST analysis (*gyrB*, *gapA*, *fruK*, *pgi*, *rpoD*, *acnB*, *gltA*) were extracted from each draft genome as well as from the three completely sequenced *P. syringae* genomes (*Pto* DC3000, *Pph* 1448a, *Psy* B728a) and from *P. fluorescens Pf-0* as an outgroup [26]–[28], [42]. Sequences were individually aligned using default parameters in ClustalX, trimmed, and concatenated. The program Mr. Bayes (http://mrbayes.csit.fsu.edu/) was used to create a Bayesian phylogeny for these sequences while parsimony-based and maximum likelihood phylogenies were created using programs within the Phylip package (http://evolution.genetics.washington.edu/phylip.html). For both parsimony and maximum likelihood, a consensus tree was created from 100 independent phylogenies. For the maximum likelihood trees, nucleotide frequencies and model were chosen using the program jModeltest (http://darwin.uvigo.es/software/modeltest.html). Similar methods were used to derive phylogenies for the *avrE*, *hopM*, *avrPto*, *avrB*, *hopF*, *hopH*, and *hopAB* families except that phylogenetic analyses were performed on protein sequences. Phylogenies built using all three methods for the MLST genes were generally congruent.

We choose to resolve problematic nodes by building amino acid sequence based trees using a database of 324 orthologous genes shared by all strains. We required reciprocal best hits (RBH), with 80% amino acid identity for greater than 80% of the length, an *e*-value <1×10^−200^ and no evidence of being on a plasmid. For each apparent ortholog, we used ProbCons, a probabilistic consistency algorithm that combines sum-of-pairs scoring and HMM-derived posterior probabilities, to produced a consensus alignment for all alleles of a gene [85]. For each gene we performed a model test to determine the best amino acid substitution model. We then concatenated all aligned sequences and, using the majority best-fit model from the individual loci, constructed a tree using RAXML (Figure S2). RAXML is a maximum likelihood-based tool for large phylogenetic trees, and was optimized for running on our computers [86], [87]. We also produced individual trees for each locus using a similar strategy, except that gene specific substitution models were used. We visually inspected and categorized a random selection of 15% of the trees to ensure consistence and to investigate discrepancies at several nodes. We observed that in cases where the tree inferred from MLST sequences differed from the protein consensus tree, the trees inferred from MLST sequences were the second common topology among trees for that gene. Individual trees are contained in Dataset S8.

### Plasmid Identification

Each draft genome sequence was surveyed with BLASTn (evalue = 1e^−5^) for the presence of structural genes associated with previously identified *P. syringae* plasmids (Table S1). Whole scaffolds/contigs containing a BLAST hit identifying that fragment as coming from a plasmid were compared to the NCBI database to identify other plasmid distinctive elements within that scaffold. Depth of coverage of Illumina reads for the entire potential plasmid scaffold/contig was compared to coverage for known housekeeping genes within each genome. Those with higher (>2×) coverage were considered ‘plasmid-like’. In strain *Pla* 107, two large (∼1 Mb total) fragments of sequence did not assemble with other contigs. Further manual assembly showed that these two genomic regions formed one large contig, and primer sets were designed to confirm manual assembly. Three primer pairs were also designed to PCR out from the ends of this potential megaplasmid, followed by Sanger based sequencing of the PCR fragments, in order to demonstrate circularization. Furthermore, six primer sets were designed to investigate the presence of this megaplasmid within closely related *P. syringae* strains.

We used the Conserved Domain Database and KEGG [88], [89] to evaluate the potential functions and metabolic pathways associated with genes harbored on the plasmids (Figure S12).

### Defining the Core and Pan Genome

We defined the core genome for all isolates using an iterative BLASTx (querying with the *Pto* DC3000 proteome, E = 10^−6^, 40% homology, 40% length hit) approach. Starting with ORFs from *Pto* DC3000 [26], we performed iterative tBLASTn versus all nucleotides of other assembled draft genomes sequentially. The other two ‘gold standard’ genomes of *Psy* B728a and *Pph* 1448a were used for the first and second iterations. During each iteration, the ‘core genome’ set – cumulatively derived to that point – was compared to an additional draft genome. In the end, ORFs from *Pto* DC3000 found within all genomes were retained as the core genome set. In addition, we performed all possible pair-wise tBLASTns, within each clade and among all clades, to determine genes unique to each isolate, clade, and sub-clade. The same procedure was performed with the genomes four *P. putida*, and three *P. fluorescens* isolates [42]–[44] to generate a ‘genus level’ core. Gene lists for the core, using the *Pto* DC3000 annotations, is found in Dataset S5, S6, S7.

To determine the pan-genome (that is, the set of genes found in at least one strain) for all isolates, we used the same iterative BLAST strategy. Only contiguous, full length ORFs (as annotated by NCBI) were used. Pseudogenes and incomplete annotations were ignored. Starting with the *P. syringae* core genome, we performed BLASTp analysis versus *Pph* 1448a, to determine which of this strain's genes were not present in the *Pseudomonas* core. These genes, combined with the core genome, are our initial pairwise “pan genome”. Subsequently this pan genome was BLASTed against all other isolates in the phylogenetic order presented in Figure 1. During each iteration, isolate genes missing (BLASTp non-hits) from the current version of the pan-genome were identified. These genes were then added to the pan-genome before the next isolate was considered. As before, we then identified clade and taxon specific “pan” genomes (pathovar unique genes are in Dataset S5, S6, S7).

### Phylogenetic Gene Gain

Starting from the most closely related isolate pair in each clade, we stepped through the phylogeny for every member of each clade, identifying the number of genes gained upon branch bifurcation, using a strategy similar to that used for the core genome above. We started by comparing genes shared by the pair of most closely related isolates (e. g. *Ptt* and *Pja*, see Figure 1) to those contained within the next closest relative (e.g. *Ppi* R6). Resulting BLASTp hits (that is, the genes shared between *Ppi* R6 and the *Ptt*-*Pja* pair) were removed, leaving the genes exclusively shared by the *Ptt*-*Pja* pair. We subsequently stepped deeper into the clade phylogeny, taking the *Ppi* R6, *Pja* and *Ptt* shared genes and comparing them to genes shared by *Psy*-*Pac* pair, thus identifying genes exclusive to the *Psy-Pac* pair and the *Ppi* R6-*Pja-Ptt* triplet respectively (Figure 2D).

### Phylo-Gene-Boost ORF Consolidation Strategy

To repair mis-annotated genes produced by the NCBI ORF annotation pipeline, we subjected all sequenced genomes to the ‘Phylo-gene-boost’ algorithm, which is similar to the “gene-boost” strategy [90]. The key difference between these two approaches is the use of phylogenetic information to inform orthology and identify suitable sequence comparisons. The NCBI annotation pipeline produces a list of continuous ORFs, along with fractured ORFs and hypothetical protein sequences. The fractured ORFs may in fact identify protein fragments from the same Cluster of Orthologous Groups (COGs, which indicate phylogenetic relatedness among the ORFs). We exploited this possibility by comparing (BLASTp e = 10^−6^, b = 1) fractured ORFs from each of the sequenced genomes to the previously assembled NCBI *P. syringae* reference genome appropriate for each clade. Resulting hits sharing a COG ID with one or more of the other hits were grouped (COG clusters). Subsequently, we aligned (ClustalW) these hits against the reference gene they matched. Locations of COG cluster ORFs with respect to the reference gene were characterized as (1) overlapping each other and the reference, (2) overlapping the reference but not each other, (3) two sequences overlapping the reference but not each other, with a linker sequence overlapping both. Group 1 sequences were concatenated, however these are potentially problematic as they may be duplication events that result in assembly errors. Group 2 sequences were concatenated as long as the unmatched region was not larger than either of the sequences. Group 3 sequences were annotated as a single ORF, retaining as much similarity to the reference protein as possible. The annotation for each isolate was updated with newly defined continuous ORFs. In some cases up to 30% of the fractured ORFs were successfully joined and annotated.

### Identification of Novel TTE Families

A subset of the sequenced strains were screened for the presence of novel TTEs using the protocol described in Chang et al [23]. Briefly, genomic libraries were created from these strains by cloning fragments into a vector upstream of promoterless GFP. These libraries were screened for the presence of active *hrp*-boxes by selecting for GFP expression using FACS sorting under conditions where the *hrpL* sigma factor was expressed. Potential TTE and *hrp*-boxes were identified after sequencing the clones. Additionally, strains pv. *maculicola* M4 (*Pma* M4, which is very closely related to *Pma*) and pv. *atrofaciens* DSM50255 (*Pat*) were screened by this method and their novel TTE sequences included in all similarity searches. TTE chimeras were also independently identified from all of the draft genomes through similarity (tBLASTn with no e-value cutoff over a significant portion of the effector) with known TTEs families or experimentally confirmed TTEs from the screen. Full length potential TTEs were cloned from at least one genome into plasmid pJC532, including sequences likely to contain the type III *hrp*-box upstream regulatory element. If *hrp* boxes could not be identified or there was a scaffold break upstream of the ORF, only the ORF sequence of the putative effector was cloned into plasmid pBAV178 where expression of an ‘Δ79AvrRpt2 fusion protein could be driven off a constitutive promoter [91]. Representatives from each putative TTE family were tested for their ability to translocate the active C-terminal fragment of AvrRpt2 to cause a hypersensitive response (HR) in Arabidopsis accession Col-0 [24]. All HR tests were performed on ∼5 week old plants and included a positive control of a known TTE cloned into either pJC532 or pBAV178 [23], [91]. The results of all tested loci are found in Dataset S9.

### Type III Effector and Phytotoxin Content

Draft genome sequences for each strain were searched by tBLASTn (at first with an with e-value cutoff of 10^−5^, but later with no cutoff) for the presence of known TTEs. This list was constructed by combining protein sequences for known *P. syringae* TTEs (http://pseudomonas-syringae.org/) with our list of novel TTEs identified from a subset of these genomes (see previous section). The potential TTE sequence was then pulled out of the draft genome sequence to the next possible stop codon and, if there was no identifiable start codon based on similarity to known TTEs, up to the earliest possible start codon after an upstream stop codon. The position of the start codon was further refined by relationship to an identifiable *hrp*-box or by comparison to other known sequences. When possible, if there was a frameshift or early stop codon that led to early protein termination (such that the locus was split into two halves that were each orthologous to a given TTE) or if there was a scaffold break disrupting a potential TTE, genomic sequences were bridged or verified by PCR-based sequencing. In some cases, the presence of TTEs could not be verified because PCR-based sequencing failed on 3 separate attempts, or because only a partial sequence of the TTE was present on a contig with no ability to bridge a gap. In some cases, there were loci in the genomes that matched by BLAST, but were significantly diverged from previously identified TTEs or were novel chimeras. In these cases, at least one subfamily member from these TTE families were cloned and tested for translocation (Dataset S9). Sequences identified as HrpL-regulated by screen or as potential TTE by similiarity, but which were not tested for translocation, are also listed in Dataset S9.

Draft genome sequences were also searched for pathways involved in construction of six well known phytotoxins associated with *P. syringae* (coronatine, phaseolotoxin, tabtoxin, syringomycin, syringopeptin, syringolin) as well as genes for ethylene production (*efe*), auxin production and modification (*iaaM*, *iaaH*, and *iaaL*) and an enzyme whose activity leads to secretion of an HR inducing factor, syringolide (*avrD*). Protein sequences for loci involved in toxin metabolic pathways in various strains were obtained from NCBI and used as a tBLASTn query on each draft genome sequence. A strain was considered to possess a toxin or gene if a majority of the protein sequences for each pathway had significant BLAST hits (<1e^−5^) with an average similarity of 80% or greater. If some, but not all, of the pathway for a particular toxin was present, the strain was considered to potentially possess the toxin.

### Analyzing the Distributions of Type III Effectors and Toxins

Within the three main clades of *P. syringae* with five or more genome sequences sampled, full length and partial TTE for each strain were assigned to bins according to prevalence within the clade. TTEs were defined as shared between strains as long as other strains within the same clade possessed the same subfamily of TTE either in the functional form or as a pseudogene/truncation. TTEs from the same subfamily, but of clearly divergent alleles within a phylogenetic group, were not classified as shared. Duplicated TTEs with no orthologous duplication in other strains were not classified as shared. For truncated or transposon disrupted TTEs, the phylogenetic node where such a change took place was identified by parsimony. If two strains within the same phylogenetic clade shared a truncated or pseudogenized TTE, positional (synteny) and sequence information was used to determine the independence of these events.

Virulence gene suites were hierarchically clustered using average linkage in Cluster 3.0 (http://bonsai.hgc.jp/~mdehoon/software/cluster/software.htm). A value of 1 was given for each potentially full-length TTE and complete phytotoxin pathways per strain. If a TTE gene or toxin biosynthetic pathway were potentially present, but unconfirmed, a value of 0.5 was given. A value of −1 was given for truncated/disrupted TTE as well as absence of a TTE. For truncated or transposon disrupted TTEs, the phylogenetic node where such a change took place was identified by parsimony. If two strains within the same phylogenetic clade shared a truncated or pseudogenized TTE, positional (synteny) and sequence information was used to determine the independence of these events.

### Pairwise Diversity Calculations for Effector Families

To calculate pairwise amino acid diversity for TTE families found within a majority of strains, TTE families were aligned using ClustalX with the default parameters. Pairwise diversity was calculated using a custom Perl script and was performed by both including deletions/insertions as divergent sites or excluding them completely. If a TTE subfamily was found to be present within multiple strains within a clade of *P. syringae*, nucleotide sequences for each member of the TTE subfamily within the clade were aligned using Revtrans 1.4 [92] with Dialign 2 alignment. The same custom Perl script was then used to calculate pairwise nucleotide diversity (π). Sites that contained insertions or deletions were not scored for these nucleotide pairwise diversity calculations. As a control, π was also calculated for the concatenated MLST gene fragments (*gyrB*, *gapA*, *fruK*, *pgi*, *rpoD*, *acnB*, *gltA*) for the same group of strains as the TTE of interest. For further investigation of *hopM1* within the group I clade, we aligned as much contiguous nucleotide sequence as possible from within five group I strains using ClustalX using default parameters without including scaffold breaks. The pairwise diversity was calculated for each site using the same custom script as above, and was averaged over 25 nucleotide windows to provide the values for Figure 6A.

### Testing the Virulence Function of hopM1_Pmp_

*hopM1* and its chaperone, *shcM* were cloned from both *Pto* DC3000 and *Pmp* into a broad host range plasmid (pBV226) for constitutive expression in *P. syringae*. Plasmids were mated into a strain [56] in which the CEL (*C*onserved *E*fector *L*ocus, including *hopM1*) was deleted. Deletion of the CEL produces a noticeable decrease in growth on Arabidopsis [55] and loss of disease symptoms of *Pto* DC3000 during growth on tomato cv. Moneymaker [56]. In Arabidopsis, we tested the ability of *hopM1* from *Pmp* to complement the virulence defect by dip inoculating of 5×10^6^ bacterial cells per mL in 10 mM MgCl_2_ into 2 week old plants. Lids were kept off of the plants for all 3 days of the experiment. Dip infection assays were performed three independent times, with 6 replicates for each genotype. Statistical analysis (ANOVA and Tukey's HSD, p≤0.05) of growth data was performed using JMP7 (SAS). The cloned fragment of *Pmp* consisting of *hopM1*, *shcM*, and the upstream *hrp* box was confirmed for HopM1 translocation using the Δ79*avrRpt2* translocation assay [23].

### *Agrobacterium*-Mediated Transient Expression Assays in *Nicotiana Benthamiana*

The tomato (*Solanum pimpinellifolium*) *Pto* gene and *avrPto* family members were cloned into pDONR207 as ORF clones without a stop codon. *avrPto* ORF clones were recombined into pGWB14 (35S∶GW∶HA) using LR clonase (Invitrogen). Similarly, *Pto* was recombined into pGWB17 (35S∶GW∶MYC). The final destination clones were moved into C58C1 (gift of Brad Day) by triparental mating. For transient expression in *N. benthamiana*, overnight cultures of Agrobacterium strains carrying *avrPto*, *Pto* or the *p19* viral suppressor (to enhance transient expression; [93]) were all washed in induction media (10 mM MgCl_2_, 10 mM MES pH 5.6, 150 uM acetosyringone) for 1 hour, diluted to OD 0.8 and mixed 1∶1∶1 and syringe inoculated. Four days later the inoculated regions were assessed for HR by visual symptoms of chlorosis/necrosis. Inoculations were performed at least three times. Expression of AvrPto and Pto was verified by Western blotting. For ion leakage assays, four 7 mm leaf disks from transient assays were taken and rinsed in distilled water for 30 min and then transferred to tubes containing 6 mL distilled water. Ion leakage was quantified over time using an Orion conductivity meter.

### *P. Syringae* Based Virulence Assays

Virulence assays using this strain on tomato were performed as described [3]. *avrPto* ORF clones were expressed in *P. syringae Pto* T1 (gift of John Rathjen) from pDLtrpGW. pDLtrpGW is a modified version of pBBR1-MCS [94] that expresses ORF clones under the constitutive *trp* promoter with a C-terminal HA tag (gifts of Derek Lundberg). Tomato plants (76R *prf3*, gift of Greg Martin) were vacuum infiltrated with OD = 0.0002 bacteria in 10 mM MgCl_2_ and 0.02% Silwet L-77. Four days after inoculation leaf discs were cored (8 replicates, each 4 cores), ground in 10 mM MgCl_2_, serially diluted and plated on KB/Rif. Statistical analysis (ANOVA and Tukey's HSD, p≤0.05) of growth data was performed using JMP7 (SAS). This experiment was performed 3 times with similar results. Newly described *avrPto* orthologs were all translocated when tested from pBAV178 (data not shown) [91].

### Accession Numbers

All genome accession numbers are listed in Table 1, at bottom. Protein accession numbers for new TTE families are listed in Table 2.

## Supporting Information

Dataset S1 **Protein sequences for all annotated ORFs within each genome for** ***Pla*** **107,** ***Pmo*** **,** ***Ppi*** **R6,** ***Pta*** **,** ***Ptt*** **,** ***Cit7*** **,** ***Pac*** **,** ***Pae*** **,** ***Pgy*** **R4,** ***Pja*** **.**
(XLSX)

Dataset S2 **Protein sequences for all annotated ORFs within each genome for** ***Pla*** **106,** ***Pmp*** **,** ***Pan*** **,** ***Pma*** **,** ***Por*** **.**
(XLSX)

Dataset S3 **Protein sequences for “broken” genes within** ***Pac*** **,** ***Pan*** **,** ***Pae*** **,** ***Ptt*** **,** ***Cit7*** **,** ***Pgy*** **R4,** ***Pja*** **,** ***Pla*** **106,** ***Pla*** **107,** ***Pmo*** **,** ***Pmp*** **,** ***Por*** **,** ***Ppi*** **R6,** ***Pta*** **.**
(XLSX)

Dataset S4 **Protein sequences for “fixed” genes within** ***Pac*** **,** ***Pan*** **,** ***Pae*** **,** ***Ptt*** **,** ***Cit7*** **,** ***Pgy*** **R4,** ***Pja*** **,** ***Pla*** **106,** ***Pla*** **107,** ***Pmo*** **,** ***Pmp*** **,** ***Por*** **,** ***Ppi*** **R6,** ***Pta*** **.**
(XLSX)

Dataset S5 **Protein sequences for the core and pan genome of** ***P. syringae*** **(** **Figure 2A,B** **), as well as core/pan genome and group specific protein sequences for groups I, II, and III (** **Figure 2D** **).**
(XLSX)

Dataset S6 **Protein sequences of group specific ORFs from each genome (** **Figure 2D** **).**
(XLSX)

Dataset S7 **Protein sequences of strain specific ORFs from each genome (** **Figure 2D** **).**
(XLSX)

Dataset S8 **A self extracting .shar file containing individual phylogenies for all 324 conserved loci which were used to generate Figure S2.** This file may be accessed by typing the file name on the command line, in the appropriate directory, without any other characters.
(SHAR)

Dataset S9 **Excel spreadsheet containing all annotated type III effectors, HrpL-regulated contigs identified by screens of the genomes, as well as unscreened or untranslocated loci.** The file contains a tab that describes all the enclosed data.
(XLS)

Figure S1 ***De Novo*** **sequencing and assembly of 14 draft** ***P. syringae*** **genomes yields a small number of relatively large scaffolds.** For each draft genome, the size of the total genome covered by scaffolds of each size is reported. Symbols for strains and phylogenetic groups are color coded as in Figure 1. For comparison, we include genome assembly metrics for both *Pto* DC3000 and *Por* 1_6 from [41].
(TIF)

Figure S2 **Consensus phylogeny for** ***P. syringae*** **based off of 324 conserved proteins.** We individually aligned 324 proteins that are conserved throughout all sequenced *P. syringae* strains, concatenated these sequences, and built a maximum likelihood phylogeny using RAXML. Strain names are color coded according to representation within MLST groups. All bootstrap values less than 100 are labeled.
(TIF)

Figure S3 **Phylo-gene-boosted ORF consolidation increases the quality of several** ***P. syringae*** **assemblies.** The white bar displays the number of continuous ORFs for each *P. syringae* isolate. The number of potential ORFS ranged from 9197 to 5706 before consolidation (open circles), but were reduced by as much as 30%. The genomes of *Pae*, *Pan*, *Pmp*, and *Pla 107* were not dramatically affected by the Phylo-gene-boost procedure, suggesting above-average assembly quality.
(TIF)

Figure S4 **Mauve alignments within group III strains.** Paired synteny alignments indicate low genome shuffling of *P. syringae* pathovars within MLST group III. Synteny of pathovars is compared in a pair wise manner (left) where each pathovar is aligned to *Pph* 1448a (top scale). Inversions are indicated by syntenic blocks placed bellow the main axis. Overall, most genomes are largely syntentic, except for *Pla* 107 where a 1Mbase of sequence – a presumed mega-plasimid – could not be aligned. Despite relatively greater sequence divergence among its members, group II (*Psy*) clade has the least genome shuffling, whereas groups I and III show qualitatively more genomic rearrangement.
(TIF)

Figure S5 **Mauve alignments within group II strains.** Paired synteny alignments indicate low genome shuffling of *P. syringae* pathovars within MLST group II. Synteny of pathovars is compared in a pair wise manner (left) where each pathovar is aligned to *Psy* B728a (top scale). Inversions are indicated by syntenic blocks placed bellow the main axis. Despite relatively greater sequence divergence among its members, group II (*Psy*) clade has the least genome shuffling, whereas groups I and III show qualitatively more genomic rearrangement.
(TIF)

Figure S6 **Mauve alignments within group I strains.** Paired synteny alignments indicate low genome shuffling of *P. syringae* pathovars within MLST group II. Synteny of pathovars is compared in a pair wise manner (left) where each pathovar is aligned to *Pto* DC3000 (top scale). Inversions are indicated by syntenic blocks placed bellow the main axis. Despite relatively greater sequence divergence among its members, group II (*Psy*) clade has the least genome shuffling, whereas groups I and III show qualitatively more genomic rearrangement.
(TIF)

Figure S7 **Characterization of the core genome for** ***P. fluorescens*** **,** ***P. putida*** **and** ***P. syringae*** **.** The core genome of these three species is functionally enriched for genes involved in biosynthesis and metabolism. Protein domain (**A**) and functional annotation (**B**) enrichment among genes in the *Pseudomonidae* core genome (see Materials and Methods) was determined using the DAVID database (http://david.abcc.ncifcrf.gov/home.jsp). DAVID compares the annotations of the submitted data (e.g. *Pseudomonidae* core genome) to annotation of a reference (“background”) set. We used the *P. syringae* core as the background. Enrichment of protein domains and functional categories is based on co-occurrence with sets of genes and their annotated functions in a gene list relative to the background. Protein domains (**A**) are predicted based on amino acid similarity to known domains. Functional classes (**B**) are as defined by DAVID [97]. Only significant and marginally significant functional categories are listed [97]. Query Enrichment (%) is the percentage of the query gene list that is in the enriched functional category.
(TIF)

Figure S8 **The** ***P. fluorescens*** **and** ***P. syringae*** **specific core genome is enriched for transport and localization.** Using the approach outlined in Figure S7, we compared protein domain (**A**) and functional annotation (**B**) enrichment within the shared *P. fluorescens* and *P. syringae* core genome. A subset of genes shared by these two species is enriched for transport and localization, compared to *Psy*, *Pfl* and *Ppt* core. Eight functional classes, designated by numbered vertical lines, are also observed among enriched functional groups in the *Pfl-Ppt-Psy* core. Numbers indicate percent enrichment of this category in the *Pfl-Ppt-Psy* core. Fewer protein domains, compared to overall and *P. syringae* specific cores, were enriched among *P. fluorescens* and *P. syringae* core (Figure S3). Query Enrichment (%) is the percentage of the query gene list that is in the enriched functional category.
(TIF)

Figure S9 ***P. syringae*** **specific core contains enrichment for localization, transport, and metabolism functional groups (similar to** ***P. fluorescens and P. syringae*** **core).** Using the approach outlined in Figure S7, we compared protein domain (**A**) and functional annotation (**B**) enrichment within the *P. syringae* specific core genome. Paralleling the *Pfl-Ppt-Psy* core, regulation of nucleotide production and carbohydrate metabolism functional groups are enriched (vertical bars indicate percent enrichment of these categories in the Pseudomonidae core). Query Enrichment (%) is the percentage of the query gene list that is in the enriched functional category.
(TIF)

Figure S10 **Characterization of group i specific core genes.** Using the approach outlined in Figure S7, we compared protein domain (**A**) and functional annotation (**B**) enrichment within the group I specific core genome. Transferase functional group is enriched among the group I specific core genes. This group is also enriched (11.76%) among *Pfl-Ppt-Psy* core functional categories (vertical bars). Five other functional groups are shared among group I core and *Pfl-Ppt-Psy* core. Group I core is the only set analyzed that contains members of the plasmid maintenance system killer functional group. Query Enrichment (%) is the percentage of the query gene list that is in the enriched functional category.
(TIF)

Figure S11 **Contig. depth.** Sequence coverage decreases drastically with increased contig length. Numbers of sequencing reads (Y-axis) covering assembled contigs (X-axis, sorted by length) for each *P. syringae* pathovar (top) were examined. Shorter contigs showed large increase in coverage when compared to longer contigs for the same *P. syringae* pathovar. These small high coverage contigs are often repetitive sequences, suggesting that multiple, nearly identical repeats are being collapsed into a signal contig. *Pae* has the highest coverage (mode of 85.67 reads per base). This value is driven by several small extremely high coverage contigs, which likely indicates that this genome has experienced a recent expansion of repeat sequences. *Pgy* R4 has the lowest coverage (mode of 10.65 reads per base). *Pla* 107 has only a few high coverage contigs (median coverage of 46.05 reads per base), the largest contig, also contains the megaplasmid sequence (red arrow), displays comparatively low coverage indicating that the megaplasmid is low copy number.
(TIF)

Figure S12 **The genome of Pla 107 harbors a recently acquired megaplasmid, pMPPla107.** (**A**) 10 sets of PCR primers were designed to confirm circular topology of the megaplasmid and to determine the presence of this megaplasmid in three related strains of *P. syringae* pv. *lachrymans* as well as *Pta* as an outgroup. (**B**) PCR confirmed that this megaplasmid is circular. Each fragment was sequenced in order to confirm the bridge. (**C**) PCR demonstrated that this megaplasmid was only present within one highly related cucumber pathogen (*Pla* N7512) and likely absent from the remaining two pv. *lachrymans* isolates. A fragment of the housekeeping gene *rpoD* was used as a PCR positive control. (**D**) Strains that possess pMPPla107 grow slightly, but significantly less (p<0.05, Tukey HSD), than strains that likely lack the megaplasmid on *Cucumis sativus* cv. Eureka. Error bars indicate 1 standard error. (**E**) Strains that possess pMP *Pla* 107 also grow more slowly on KB rif plates than strains that lack the megaplasmid. (**F**) We searched the Conserved Domain Database (CDD, shown in figure) as well as the KEGG database (data not shown) in order to identify whole pathways that were present on the megaplasmid. This approach surveys domains within predicted proteins and compares this set to functional families corresponding to known pathways. The 15 most abundant categories are represented along with the percentage of proteins within the megaplasmid containing these domains. None of the predicted pathways appears complete.
(TIF)

Figure S13 **Associations between virulence genes or pathways and similarity of strain repertoires.** Strains (color coded by phylogenetic group according to figure 1) and virulence factors (including TTE families and phytotoxin pathways) were hierarchically clustered based on the virulence gene repertoires of each strain. In only a small number of cases was there enough information to see associations among virulence factors (i.e. *efe* and *avrRps4*) above and beyond operon linkage or phylogeny. Interestingly, and differing from phylogenies based on sequences of core genes, *Pan* clusters more closely to group III strains, indicating similarity of virulence gene repertoires. *Pmp* clusters outside of group I, indicating divergence of its repertoires from that group. *Ppi* R6 clusters outside of other group II strains, demonstrating differences in the virulence gene repertoires. Yellow boxes indicate presence, muted yellow boxes indicate unconfirmed presence, and gray boxes indicate absence/pseudogenization/truncation.
(TIF)

Figure S14 **Highly divergent TTE families are horizontally transferred at high rates.** Bayesian (left) and Parsimony based (right) phylogenies were constructed for three of the most diverse TTE families (**A**) *hopAB* (**B**) *hopF* (**C**) *avrB*. Strain names are color coded by phylogenetic group, as in Figure 1, except where phylogenetic position was unknown (in black). All nodes with posterior probabilities (A) or bootstrap support (B) below 0.95/95 were labeled on the phylogenies.
(TIF)

Figure S15 **Distantly related** ***P. syringae*** **pv.** ***lachrymans*** **strains carry divergent TTE repertoires.** (**A**) TTE repertoires were compared for *Pla* 106 and *Pla* 107, which are both isolated from diseased cucumbers but are members of different *P. syringae* phylogenetic groups. TTE families found within a majority (>10) of all sequenced *P. syringae* strains are shown in white, while those found in <10 genomes are listed in black. TTE disrupted by either truncation or insertion elements are listed in red. (B) A representative growth curve from two independent experiments (3 replicates for each genotype) on *Cucumis sativus* cv. Eureka is shown. Orange bars represent bacterial counts in inoculum while blue bars represent bacterial counts after 6 days of growth *in planta*. Dashed line represents estimated bacterial count *in planta* at day 0. Error bars indicate 1 standard error.
(TIF)

Figure S16 **AvrPto alignment; expression of AvrPto/Pto in transient assays; GINP loop diversity.** (**A**) ClustalW alignment of the AvrPto superfamily. Conserved residues are highlighted in blue. The GINP loop is highlighted in red. (**B**) Western blotting of Agrobacterium-*N. benthamiana* transient assay. AvrPto orthologs are HA epitope tagged, while Pto is tagged with the c-myc epitope. Ponceau staining reflects overall protein loading. Unrecognized AvrPto orthologs are expressed at least as well as AvrPto, indicating that lack of avirulence is not merely due to a lack of expression. (**C**) GINP loop region of the AvrPto/Pto co-crystal, orientation as in [58]. AvrPto is in orange, Pto purple. (**D**) AvrPto*_Pgy_*_R4_ sequence modeled onto the AvrPto crystal structure, I85 and G95 are shown in cyan. (**E**) AvrPto crystal structure with known loss of avirulence mutations S94P, I96T and G99V shown in red [64]. (**F**) AvrPto*_Pmo_* sequence modeled onto AvrPto crystal structure, S95 shown in blue. Images (**C**)–(**F**) generated using the PyMOL software package [98].
(TIF)

Table S1 **A majority of** ***P. syringae*** **strains harbor endogenous plasmids.** Putative plasmid sequences were identified through BLAST searches against fourteen common and typically plasmid localized sequences (horizontal). For each draft genome that contains putative plasmids (vertical), the average coverage level and standard deviation of coverage (in parentheses) over the contig containing that fragment is reported. As a control, the average coverage over *gyrB*, *gapA*, and *rpoD* is reported as an estimate for chromosomal coverage levels. Genomic data for *Pmp* has the highest difference between the average chromosome coverage (24.5) and putative plasmid sequence coverage (359.0 - *MobA*).
(TIF)

Table S2 **A subset of** ***P. syringae*** **strains with draft genome sequences were screened for novel TTE families to near saturation.** The number of contigs containing *hrpL*-regulated TTSS and common effector genes recovered in our functional screen is given, as are data for strains from the previously reported screen [23].
(TIF)

Table S3 **TTE protein families display wide ranging values for amino acid divergence.** Pairwise amino acid divergence was calculated for all TTE families represented in a majority of strains. Divergence was calculated both by including each positional gap in the sequence as a divergent site or by excluding gaps altogether.
(TIF)
