# Supplementary figures and images for: Dynamic Evolution of Pathogenicity Revealed by Sequencing and Comparative Genomics of 19 Pseudomonas syringae Isolates
Source: PLoS Pathog. 2011 Jul 14;7(7):e1002132. doi: 10.1371/journal.ppat.1002132 (PMC3136466; doi:10.1371/journal.ppat.1002132)

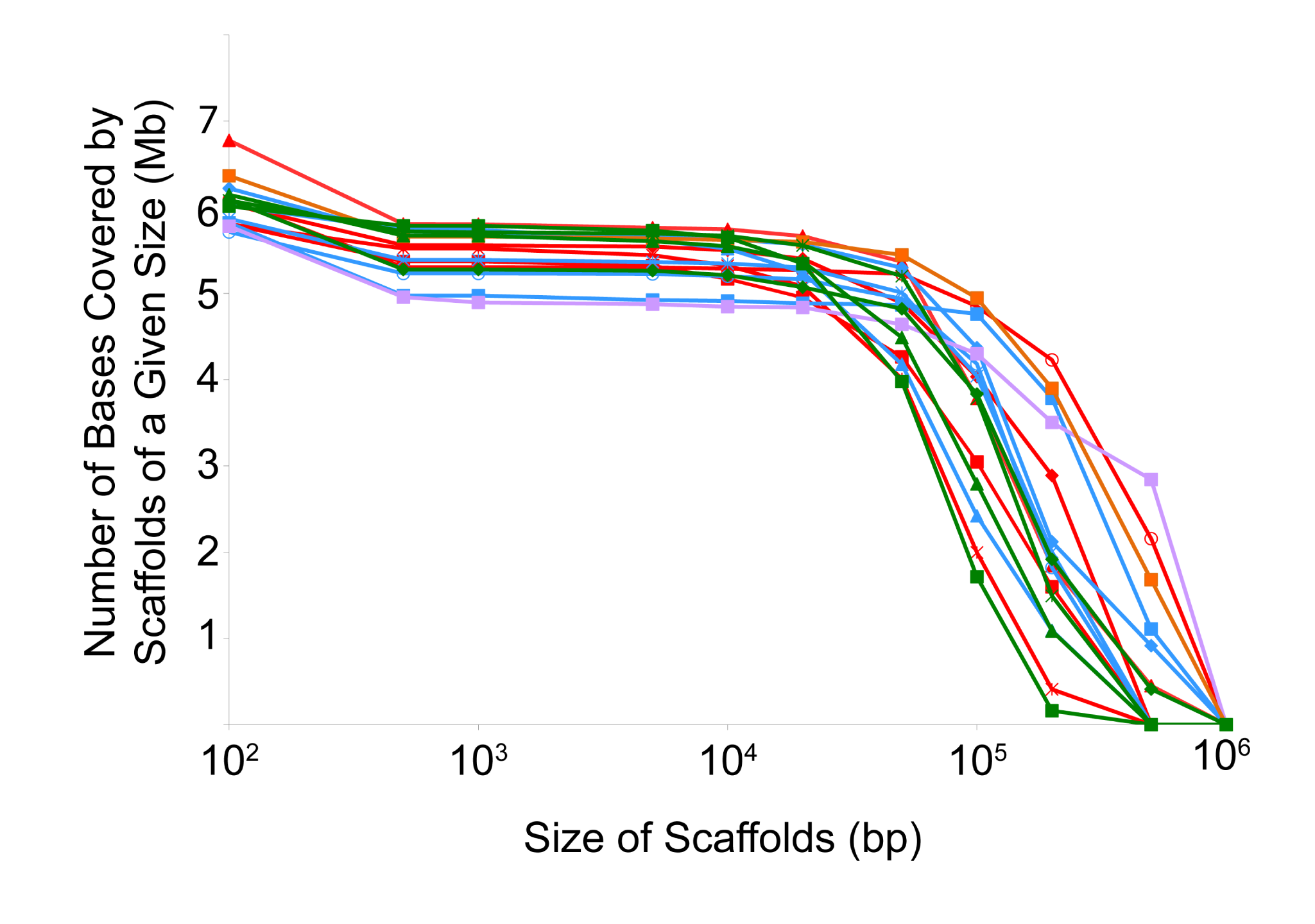

Supplement: Figure S1 — De Novo sequencing and assembly of 14 draft P. syringae genomes yields a small number of relatively large scaffolds. For each draft genome, the size of the total genome covered by scaffolds of each size is reported. Symbols for strains and phylogenetic groups are color coded as in Figure 1. For comparison, we include genome assembly metrics for both Pto DC3000 and Por 1_6 from [41]. (TIF) [file ppat.1002132.s010.tif]

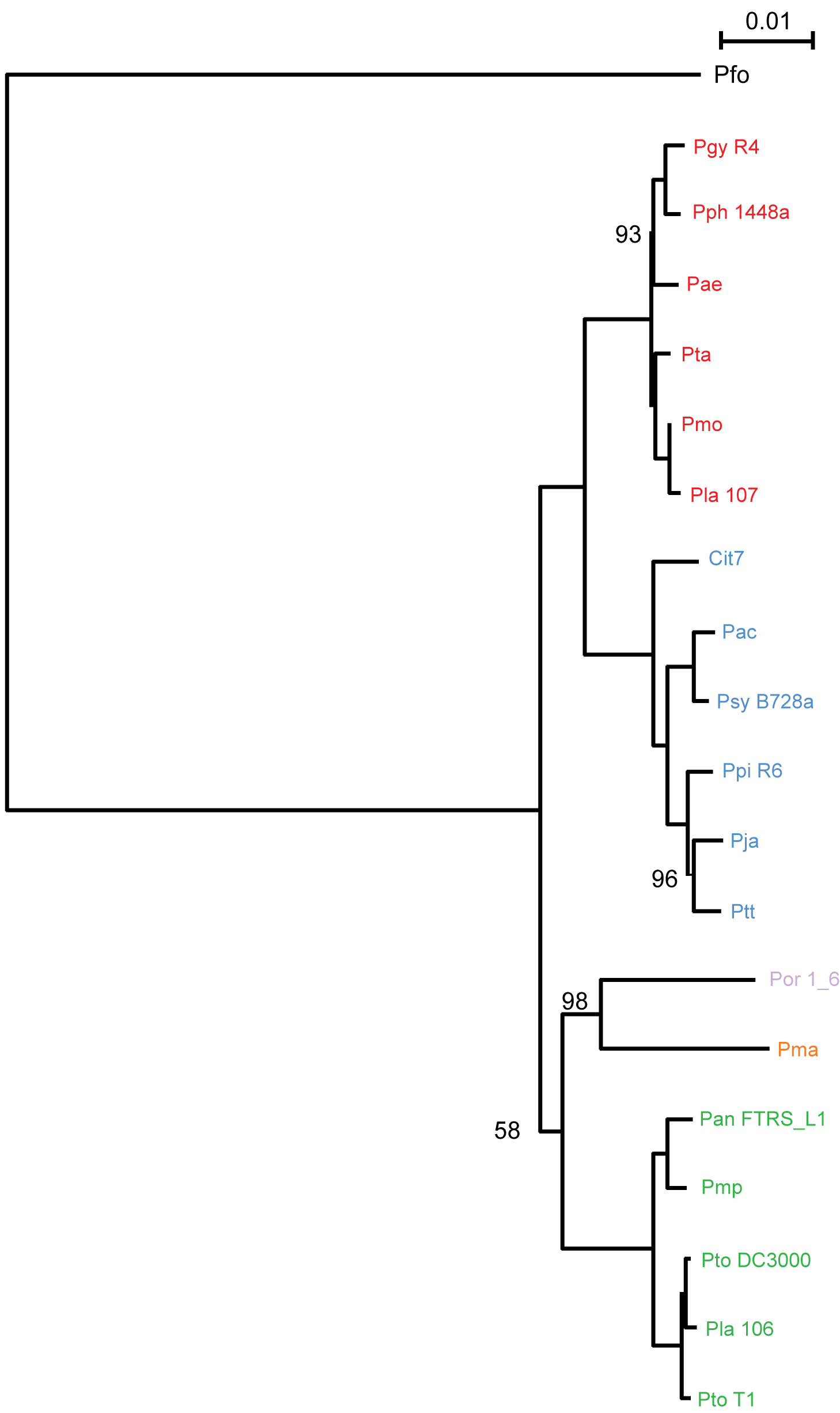

Supplement: Figure S2 — Consensus phylogeny for P. syringae based off of 324 conserved proteins. We individually aligned 324 proteins that are conserved throughout all sequenced P. syringae strains, concatenated these sequences, and built a maximum likelihood phylogeny using RAXML. Strain names are color coded according to representation within MLST groups. All bootstrap values less than 100 are labeled. (TIF) [file ppat.1002132.s011.tif]

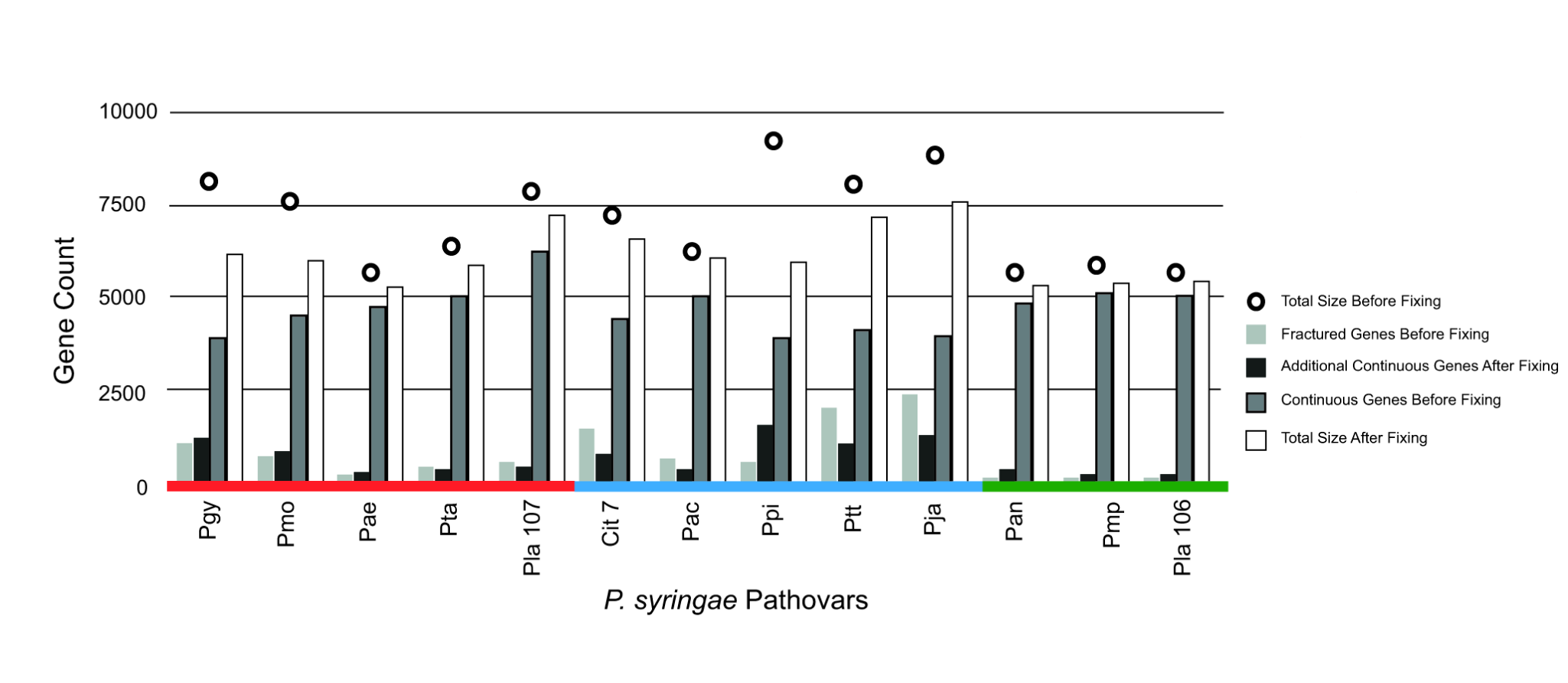

Supplement: Figure S3 — Phylo-gene-boosted ORF consolidation increases the quality of several P. syringae assemblies. The white bar displays the number of continuous ORFs for each P. syringae isolate. The number of potential ORFS ranged from 9197 to 5706 before consolidation (open circles), but were reduced by as much as 30%. The genomes of Pae, Pan, Pmp, and Pla 107 were not dramatically affected by the Phylo-gene-boost procedure, suggesting above-average assembly quality. (TIF) [file ppat.1002132.s012.tif]

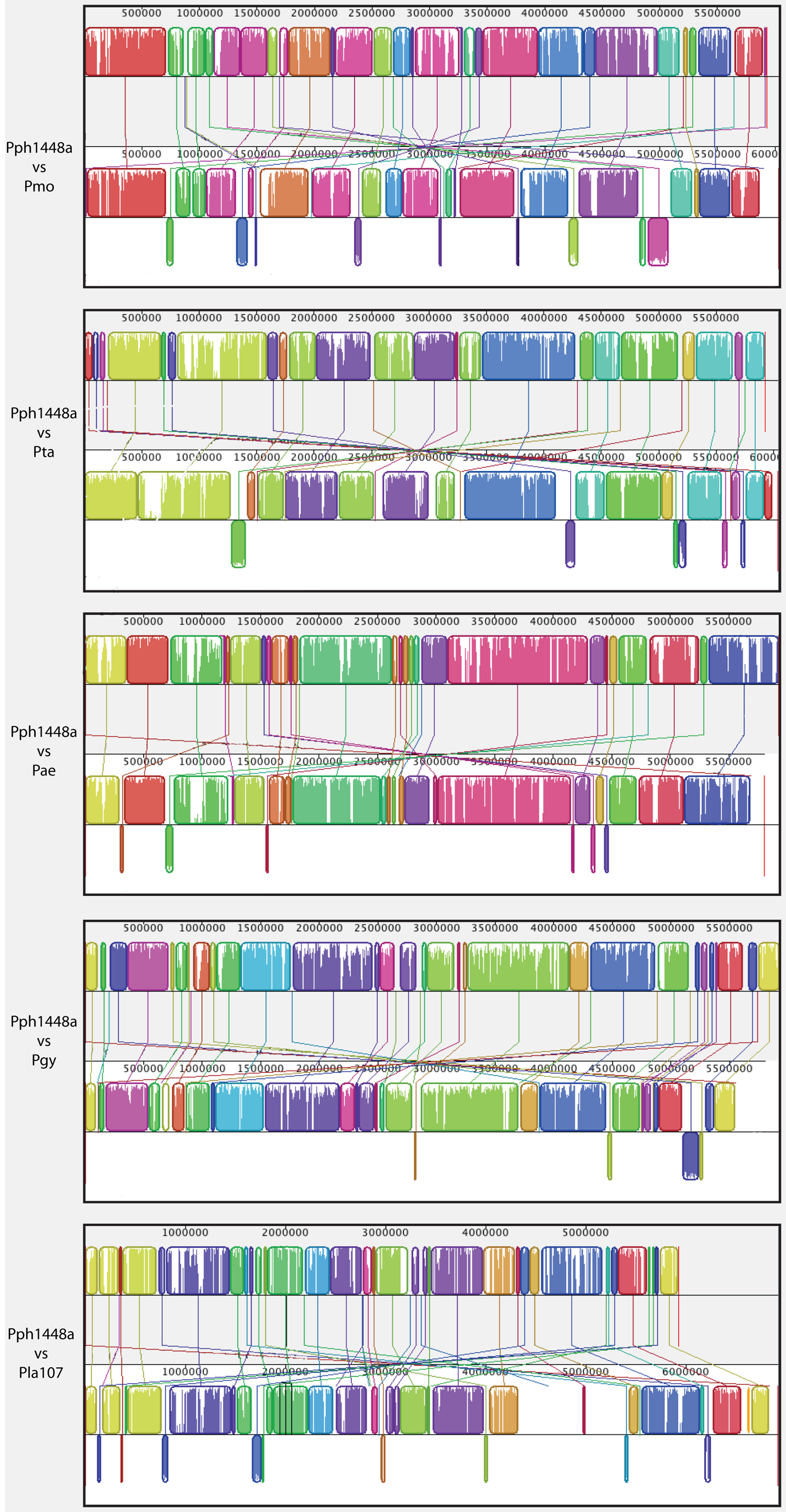

Supplement: Figure S4 — Mauve alignments within group III strains. Paired synteny alignments indicate low genome shuffling of P. syringae pathovars within MLST group III. Synteny of pathovars is compared in a pair wise manner (left) where each pathovar is aligned to Pph 1448a (top scale). Inversions are indicated by syntenic blocks placed bellow the main axis. Overall, most genomes are largely syntentic, except for Pla 107 where a 1Mbase of sequence – a presumed mega-plasimid – could not be aligned. Despite relatively greater sequence divergence among its members, group II (Psy) clade has the least genome shuffling, whereas groups I and III show qualitatively more genomic rearrangement. (TIF) [file ppat.1002132.s013.tif]

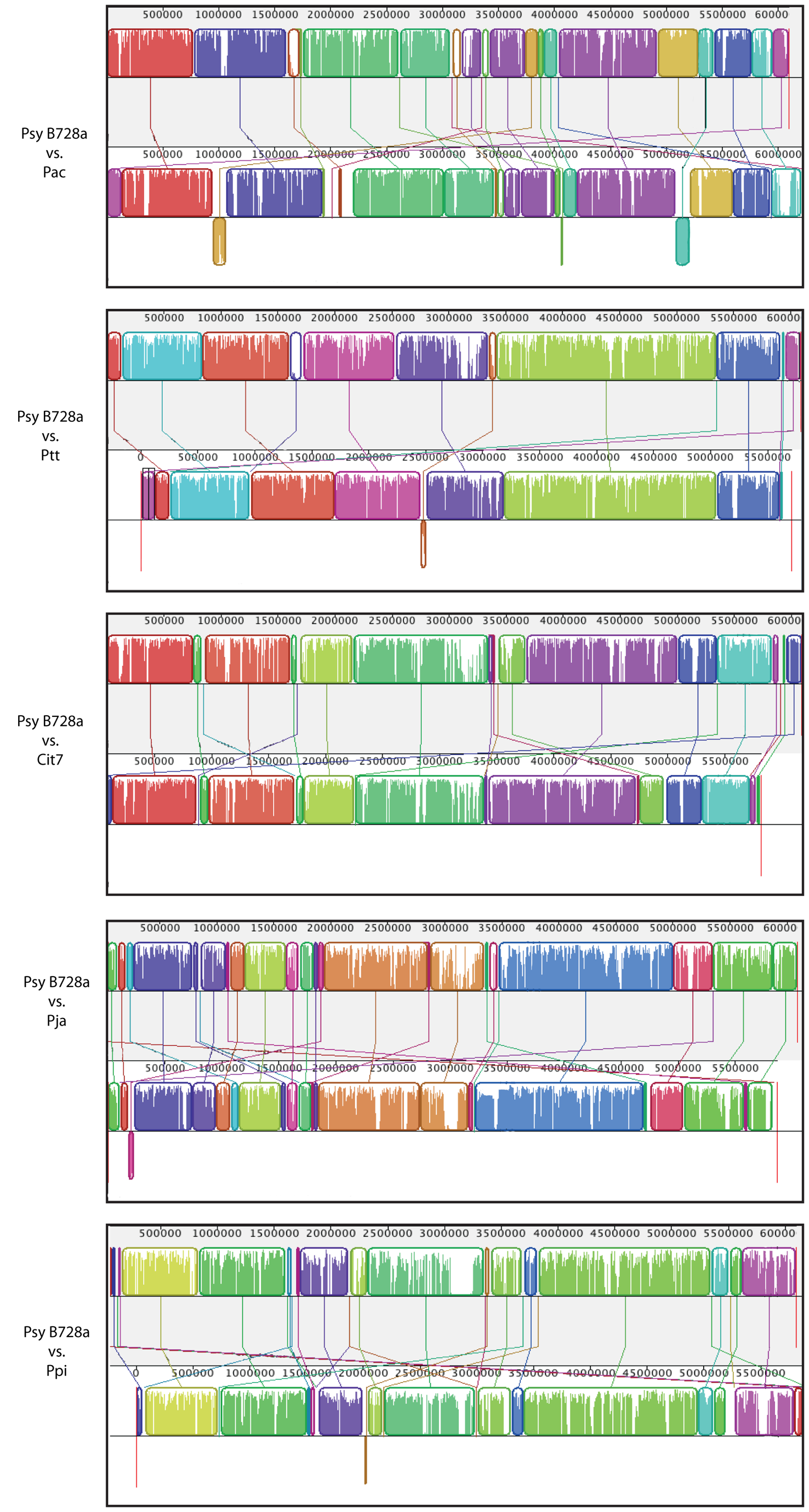

Supplement: Figure S5 — Mauve alignments within group II strains. Paired synteny alignments indicate low genome shuffling of P. syringae pathovars within MLST group II. Synteny of pathovars is compared in a pair wise manner (left) where each pathovar is aligned to Psy B728a (top scale). Inversions are indicated by syntenic blocks placed bellow the main axis. Despite relatively greater sequence divergence among its members, group II (Psy) clade has the least genome shuffling, whereas groups I and III show qualitatively more genomic rearrangement. (TIF) [file ppat.1002132.s014.tif]

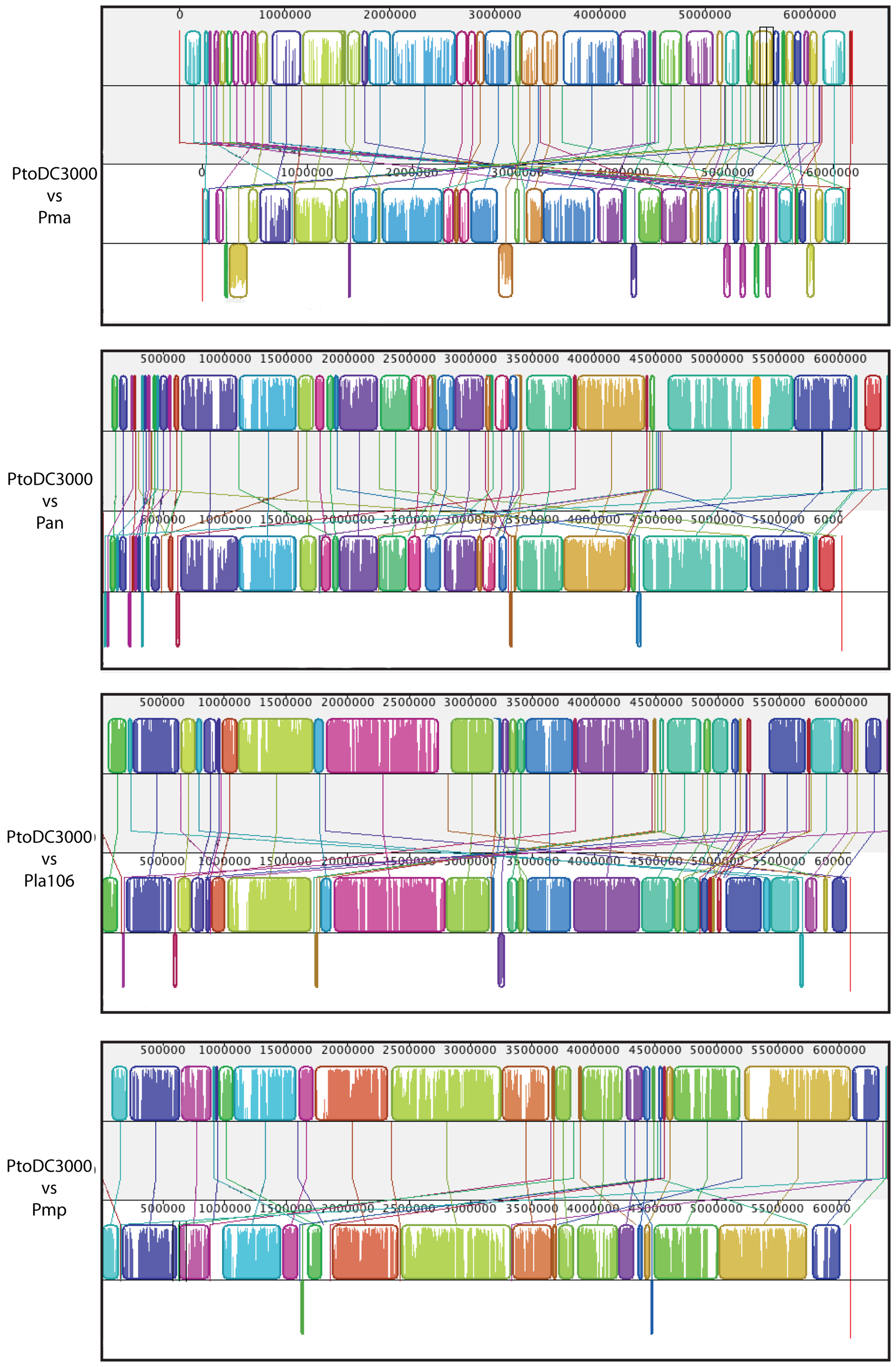

Supplement: Figure S6 — Mauve alignments within group I strains. Paired synteny alignments indicate low genome shuffling of P. syringae pathovars within MLST group II. Synteny of pathovars is compared in a pair wise manner (left) where each pathovar is aligned to Pto DC3000 (top scale). Inversions are indicated by syntenic blocks placed bellow the main axis. Despite relatively greater sequence divergence among its members, group II (Psy) clade has the least genome shuffling, whereas groups I and III show qualitatively more genomic rearrangement. (TIF) [file ppat.1002132.s015.tif]

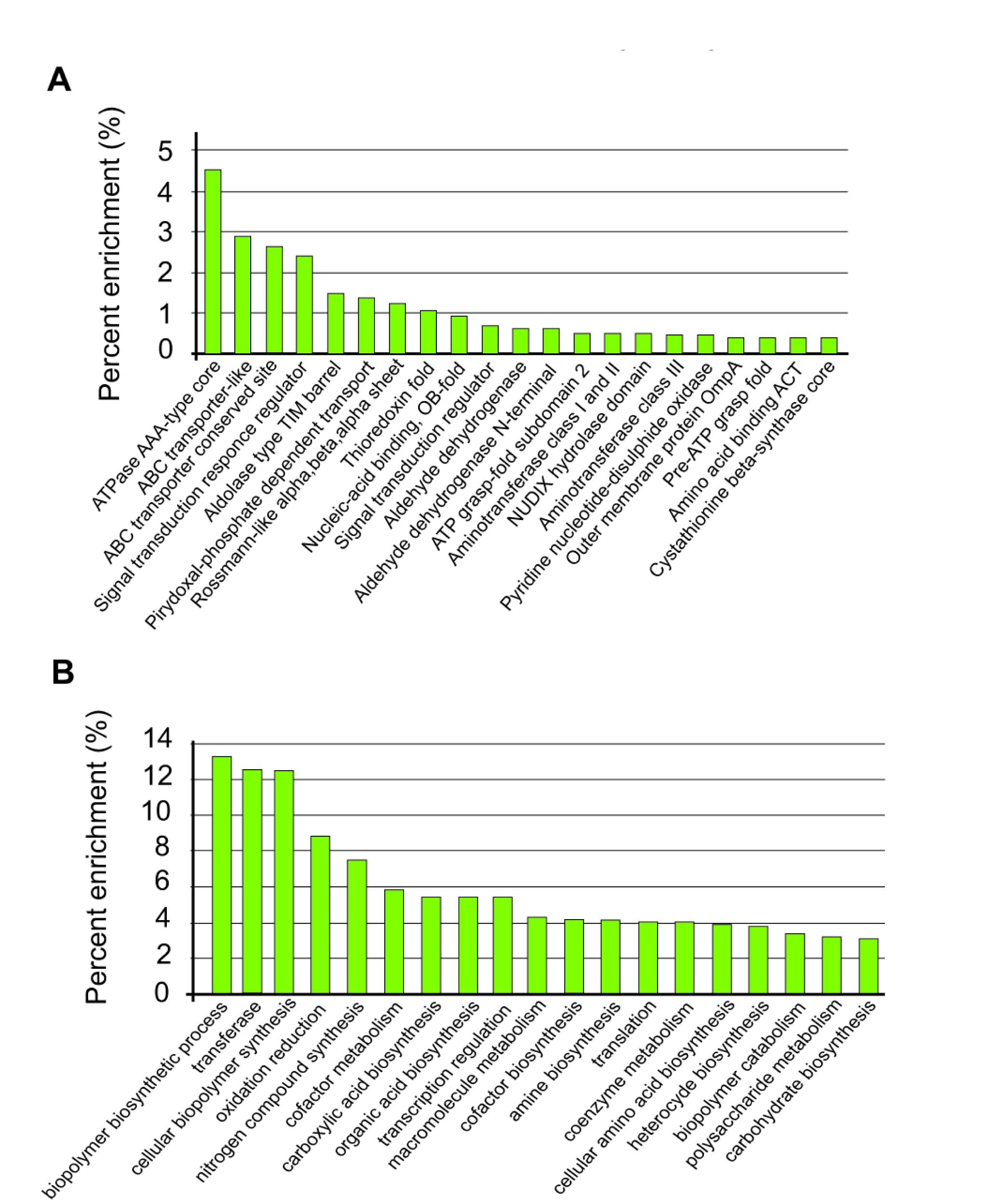

Supplement: Figure S7 — Characterization of the core genome for P. fluorescens , P. putida and P. syringae . The core genome of these three species is functionally enriched for genes involved in biosynthesis and metabolism. Protein domain (A) and functional annotation (B) enrichment among genes in the Pseudomonidae core genome (see Materials and Methods) was determined using the DAVID database (http://david.abcc.ncifcrf.gov/home.jsp). DAVID compares the annotations of the submitted data (e.g. Pseudomonidae core genome) to annotation of a reference (“background”) set. We used the P. syringae core as the background. Enrichment of protein domains and functional categories is based on co-occurrence with sets of genes and their annotated functions in a gene list relative to the background. Protein domains (A) are predicted based on amino acid similarity to known domains. Functional classes (B) are as defined by DAVID [97]. Only significant and marginally significant functional categories are listed [97]. Query Enrichment (%) is the percentage of the query gene list that is in the enriched functional category. (TIF) [file ppat.1002132.s016.tif]

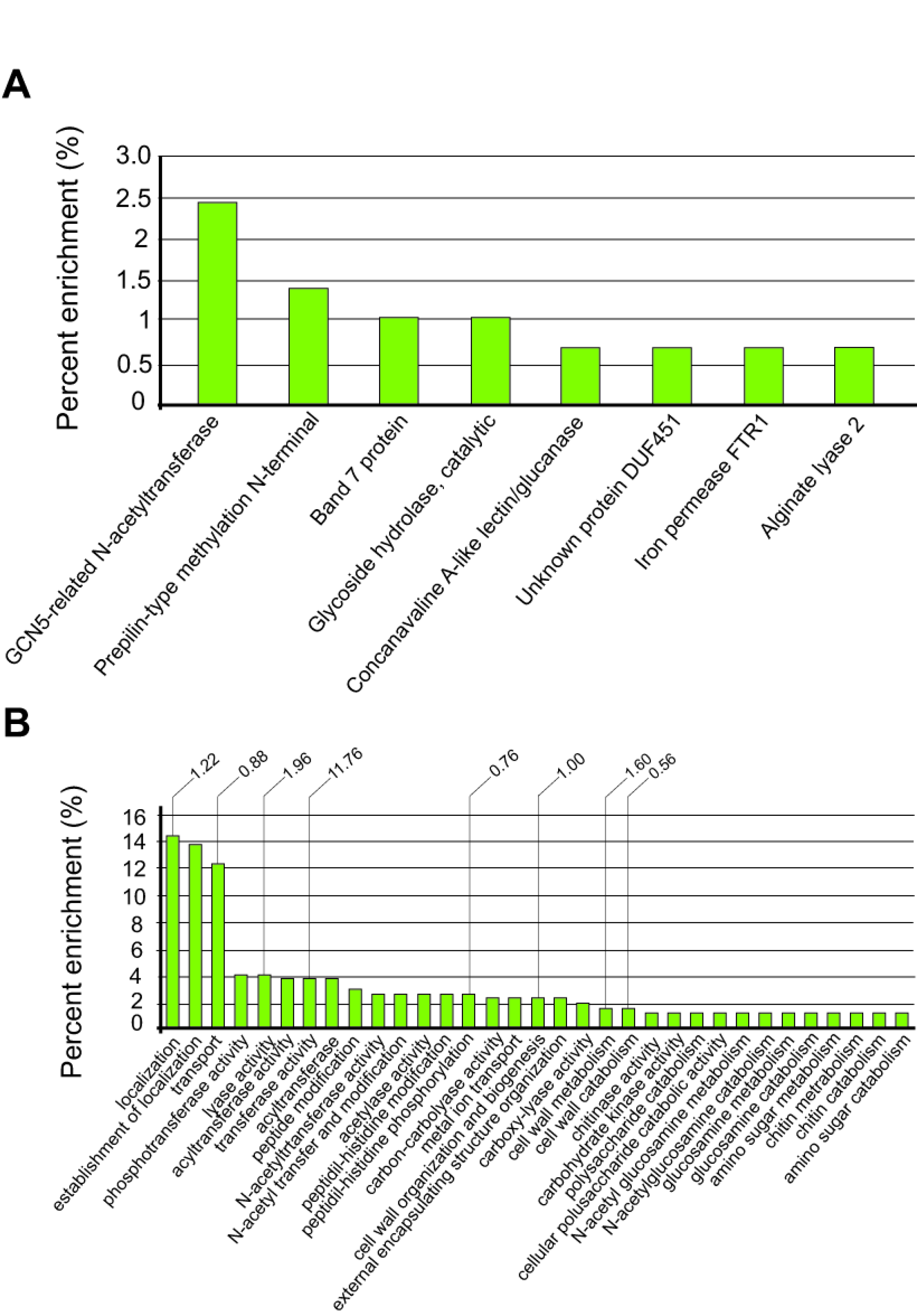

Supplement: Figure S8 — The P. fluorescens and P. syringae specific core genome is enriched for transport and localization. Using the approach outlined in Figure S7, we compared protein domain (A) and functional annotation (B) enrichment within the shared P. fluorescens and P. syringae core genome. A subset of genes shared by these two species is enriched for transport and localization, compared to Psy, Pfl and Ppt core. Eight functional classes, designated by numbered vertical lines, are also observed among enriched functional groups in the Pfl-Ppt-Psy core. Numbers indicate percent enrichment of this category in the Pfl-Ppt-Psy core. Fewer protein domains, compared to overall and P. syringae specific cores, were enriched among P. fluorescens and P. syringae core (Figure S3). Query Enrichment (%) is the percentage of the query gene list that is in the enriched functional category. (TIF) [file ppat.1002132.s017.tif]

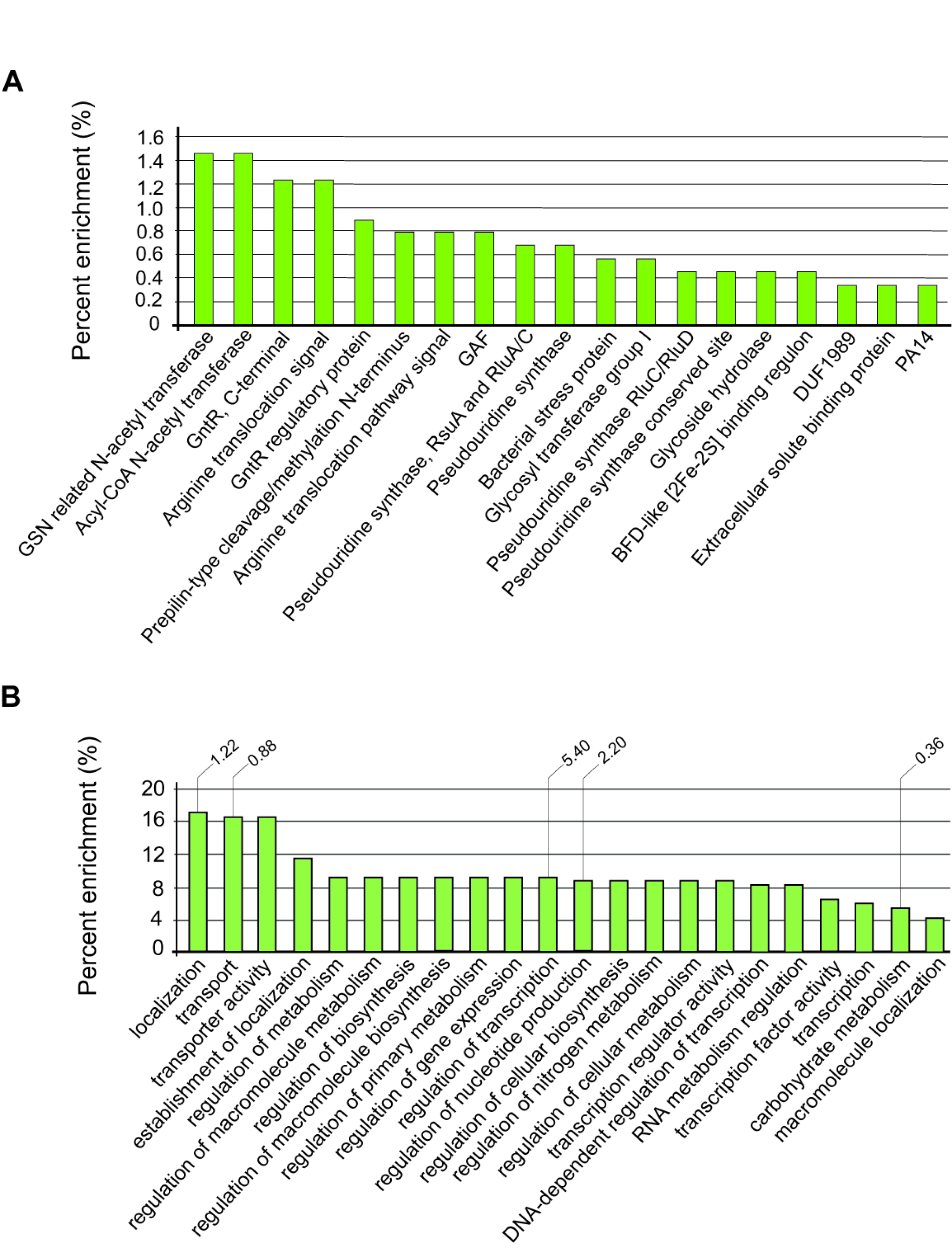

Supplement: Figure S9 — P. syringae specific core contains enrichment for localization, transport, and metabolism functional groups (similar to P. fluorescens and P. syringae core). Using the approach outlined in Figure S7, we compared protein domain (A) and functional annotation (B) enrichment within the P. syringae specific core genome. Paralleling the Pfl-Ppt-Psy core, regulation of nucleotide production and carbohydrate metabolism functional groups are enriched (vertical bars indicate percent enrichment of these categories in the Pseudomonidae core). Query Enrichment (%) is the percentage of the query gene list that is in the enriched functional category. (TIF) [file ppat.1002132.s018.tif]

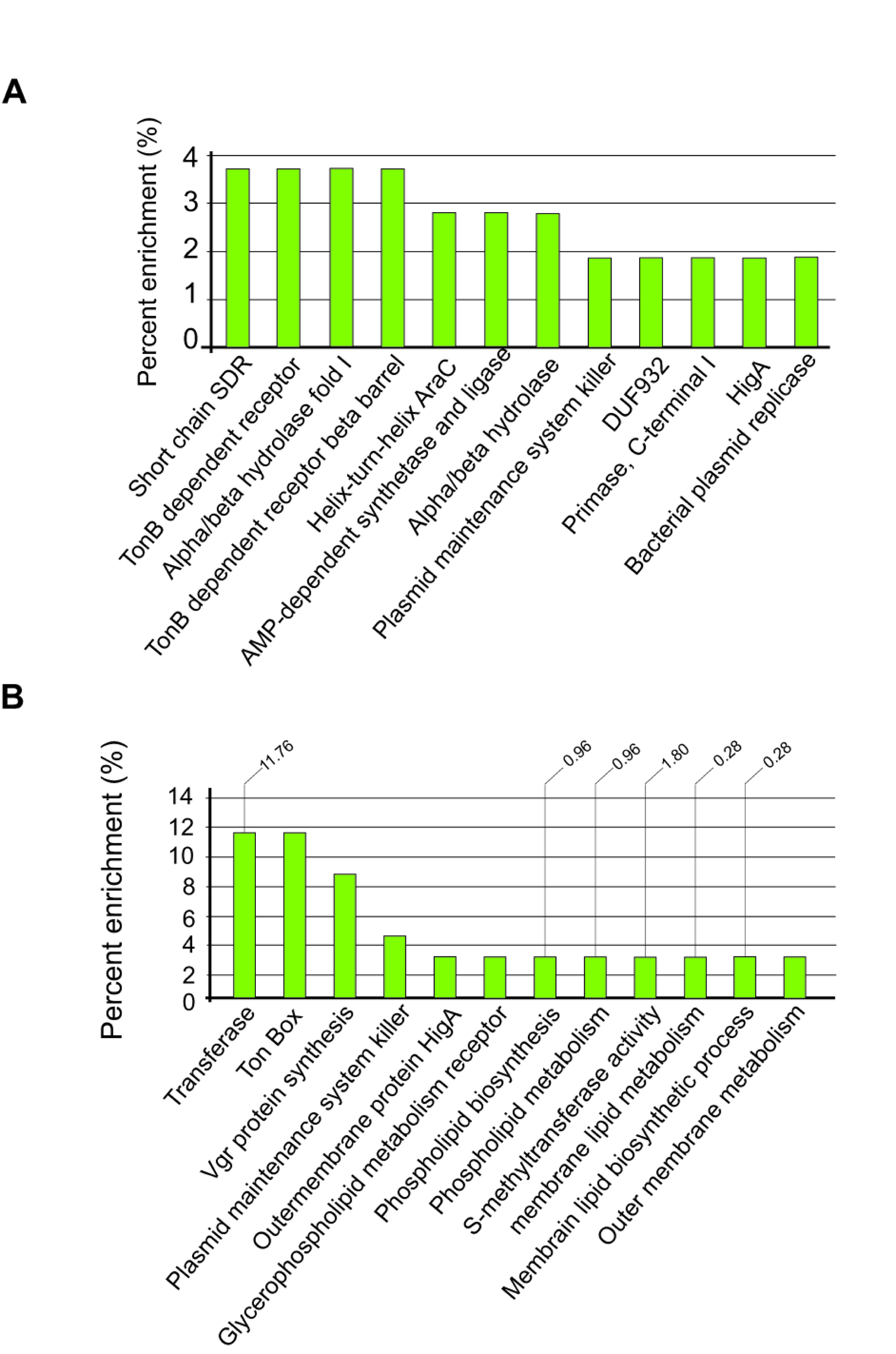

Supplement: Figure S10 — Characterization of group i specific core genes. Using the approach outlined in Figure S7, we compared protein domain (A) and functional annotation (B) enrichment within the group I specific core genome. Transferase functional group is enriched among the group I specific core genes. This group is also enriched (11.76%) among Pfl-Ppt-Psy core functional categories (vertical bars). Five other functional groups are shared among group I core and Pfl-Ppt-Psy core. Group I core is the only set analyzed that contains members of the plasmid maintenance system killer functional group. Query Enrichment (%) is the percentage of the query gene list that is in the enriched functional category. (TIF) [file ppat.1002132.s019.tif]

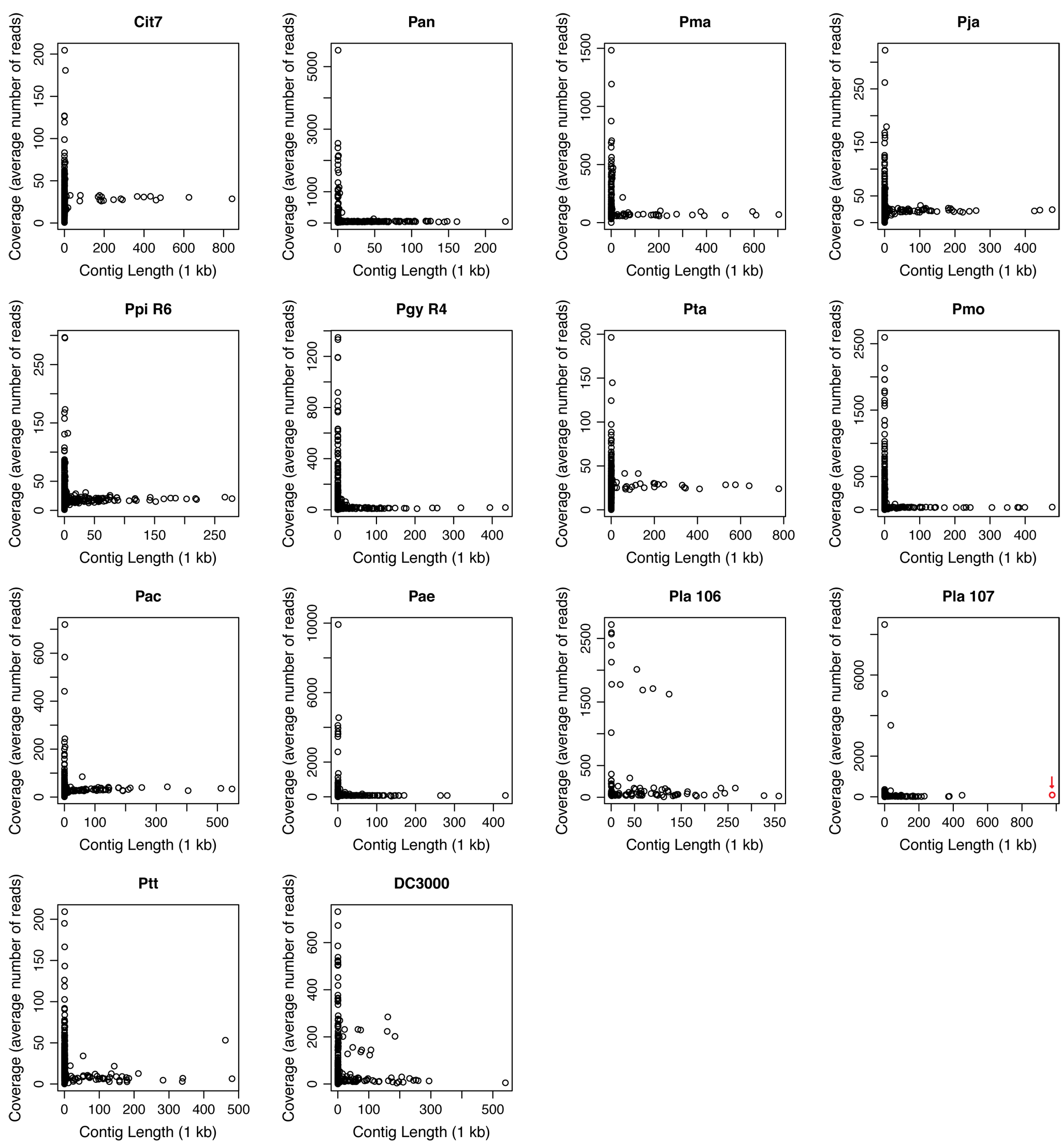

Supplement: Figure S11 — Contig. depth. Sequence coverage decreases drastically with increased contig length. Numbers of sequencing reads (Y-axis) covering assembled contigs (X-axis, sorted by length) for each P. syringae pathovar (top) were examined. Shorter contigs showed large increase in coverage when compared to longer contigs for the same P. syringae pathovar. These small high coverage contigs are often repetitive sequences, suggesting that multiple, nearly identical repeats are being collapsed into a signal contig. Pae has the highest coverage (mode of 85.67 reads per base). This value is driven by several small extremely high coverage contigs, which likely indicates that this genome has experienced a recent expansion of repeat sequences. Pgy R4 has the lowest coverage (mode of 10.65 reads per base). Pla 107 has only a few high coverage contigs (median coverage of 46.05 reads per base), the largest contig, also contains the megaplasmid sequence (red arrow), displays comparatively low coverage indicating that the megaplasmid is low copy number. (TIF) [file ppat.1002132.s020.tif]

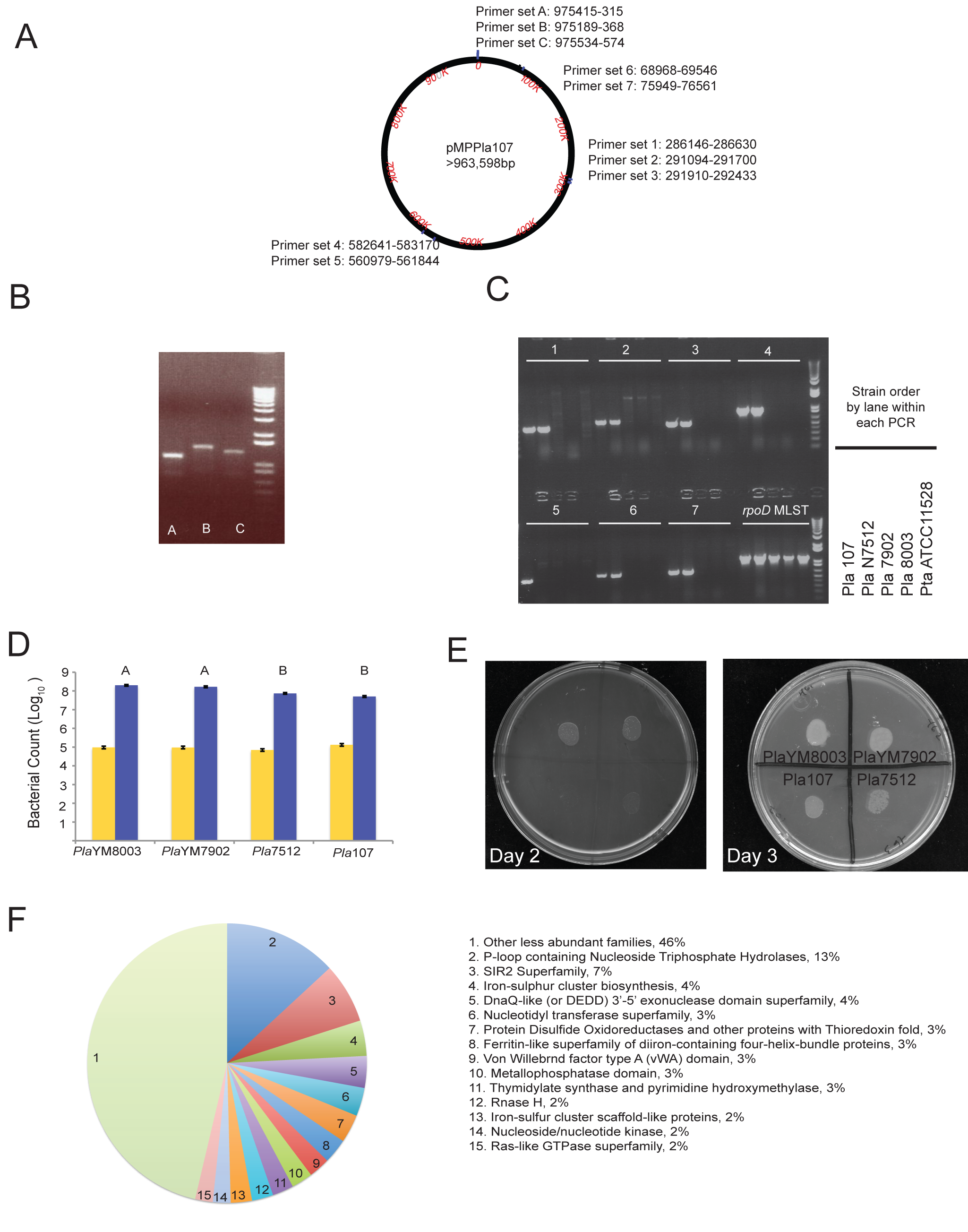

Supplement: Figure S12 — The genome of Pla 107 harbors a recently acquired megaplasmid, pMPPla107. (A) 10 sets of PCR primers were designed to confirm circular topology of the megaplasmid and to determine the presence of this megaplasmid in three related strains of P. syringae pv. lachrymans as well as Pta as an outgroup. (B) PCR confirmed that this megaplasmid is circular. Each fragment was sequenced in order to confirm the bridge. (C) PCR demonstrated that this megaplasmid was only present within one highly related cucumber pathogen (Pla N7512) and likely absent from the remaining two pv. lachrymans isolates. A fragment of the housekeeping gene rpoD was used as a PCR positive control. (D) Strains that possess pMPPla107 grow slightly, but significantly less (p<0.05, Tukey HSD), than strains that likely lack the megaplasmid on Cucumis sativus cv. Eureka. Error bars indicate 1 standard error. (E) Strains that possess pMP Pla 107 also grow more slowly on KB rif plates than strains that lack the megaplasmid. (F) We searched the Conserved Domain Database (CDD, shown in figure) as well as the KEGG database (data not shown) in order to identify whole pathways that were present on the megaplasmid. This approach surveys domains within predicted proteins and compares this set to functional families corresponding to known pathways. The 15 most abundant categories are represented along with the percentage of proteins within the megaplasmid containing these domains. None of the predicted pathways appears complete. (TIF) [file ppat.1002132.s021.tif]

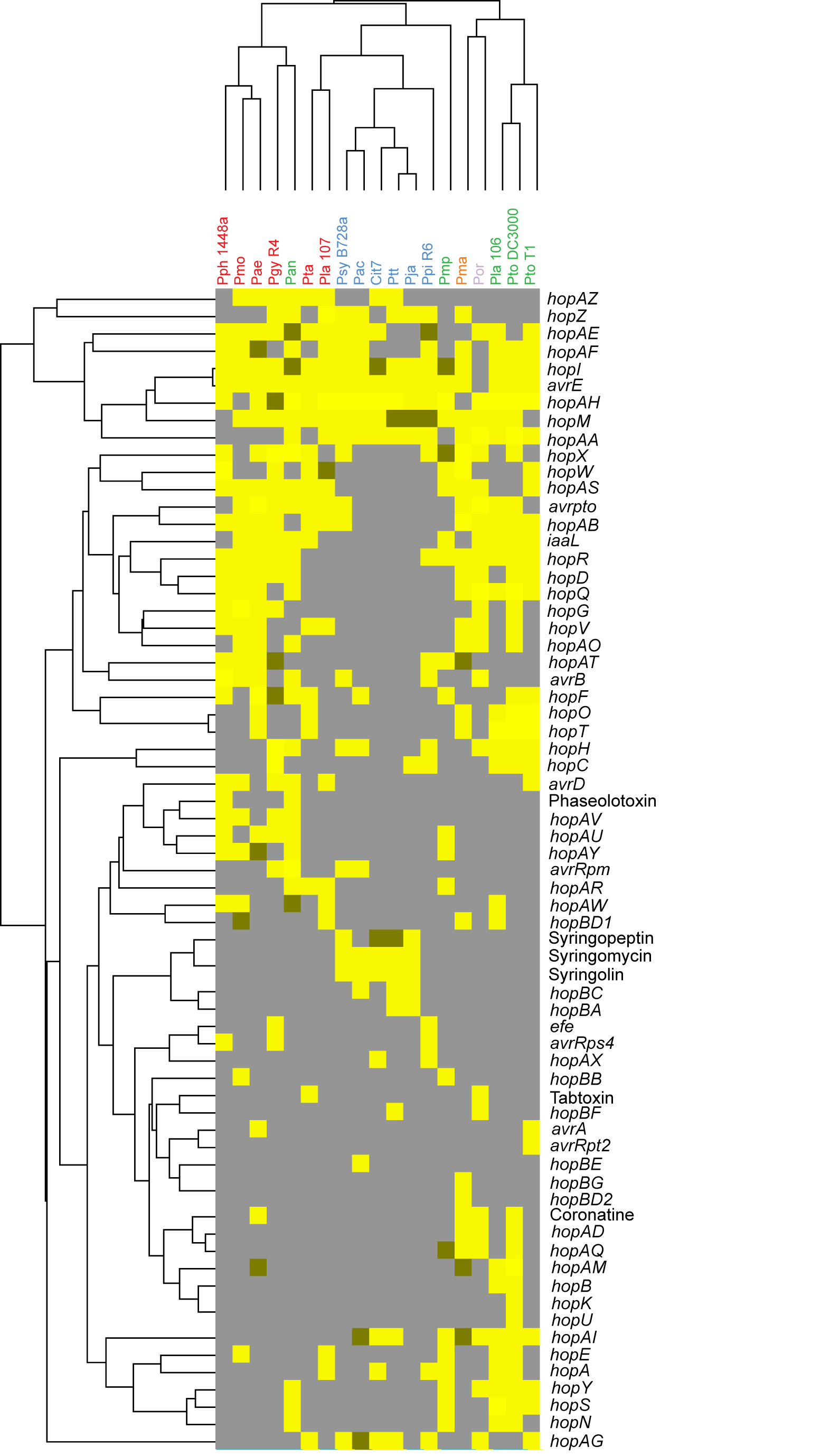

Supplement: Figure S13 — Associations between virulence genes or pathways and similarity of strain repertoires. Strains (color coded by phylogenetic group according to figure 1) and virulence factors (including TTE families and phytotoxin pathways) were hierarchically clustered based on the virulence gene repertoires of each strain. In only a small number of cases was there enough information to see associations among virulence factors (i.e. efe and avrRps4) above and beyond operon linkage or phylogeny. Interestingly, and differing from phylogenies based on sequences of core genes, Pan clusters more closely to group III strains, indicating similarity of virulence gene repertoires. Pmp clusters outside of group I, indicating divergence of its repertoires from that group. Ppi R6 clusters outside of other group II strains, demonstrating differences in the virulence gene repertoires. Yellow boxes indicate presence, muted yellow boxes indicate unconfirmed presence, and gray boxes indicate absence/pseudogenization/truncation. (TIF) [file ppat.1002132.s022.tif]

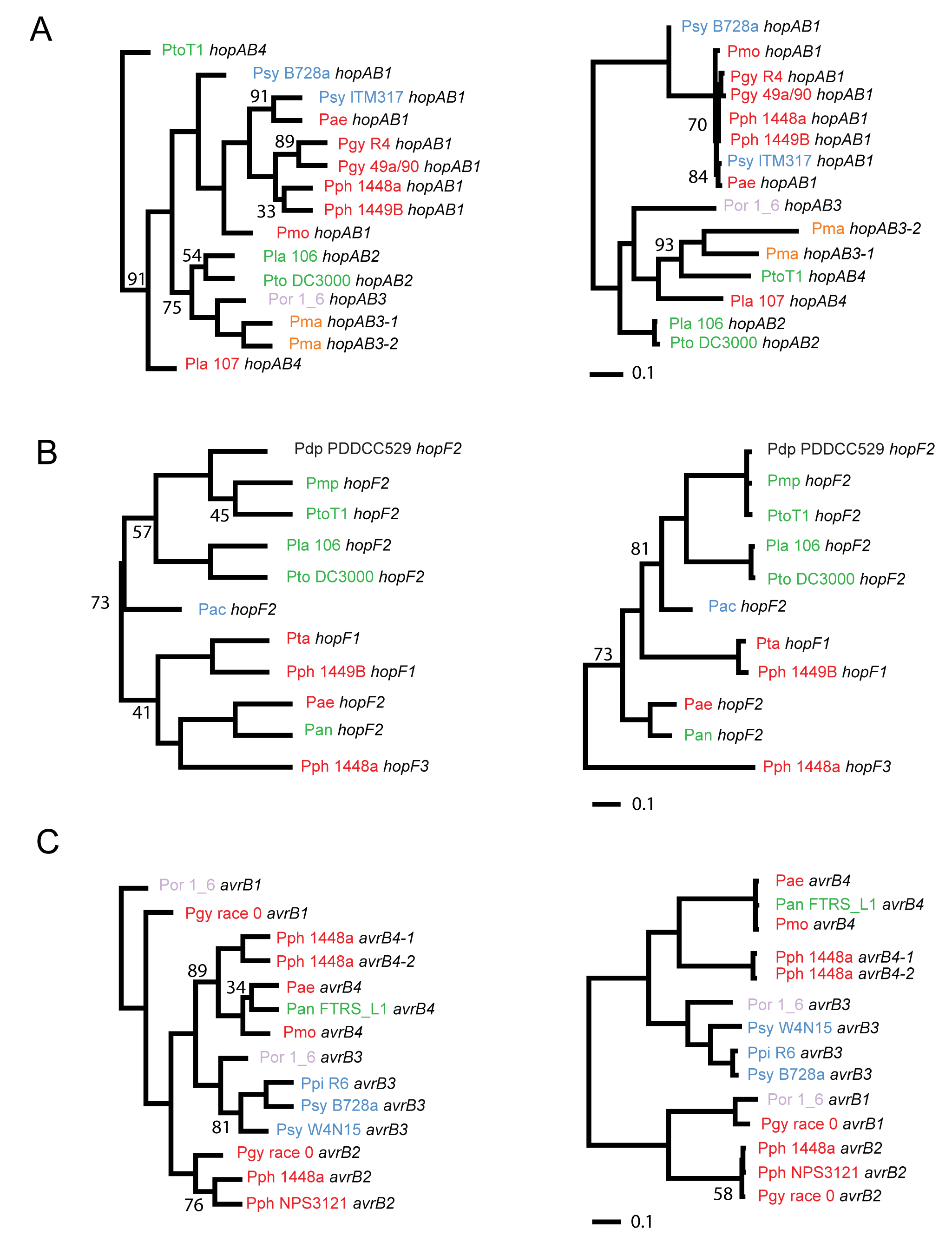

Supplement: Figure S14 — Highly divergent TTE families are horizontally transferred at high rates. Bayesian (left) and Parsimony based (right) phylogenies were constructed for three of the most diverse TTE families (A) hopAB (B) hopF (C) avrB. Strain names are color coded by phylogenetic group, as in Figure 1, except where phylogenetic position was unknown (in black). All nodes with posterior probabilities (A) or bootstrap support (B) below 0.95/95 were labeled on the phylogenies. (TIF) [file ppat.1002132.s023.tif]

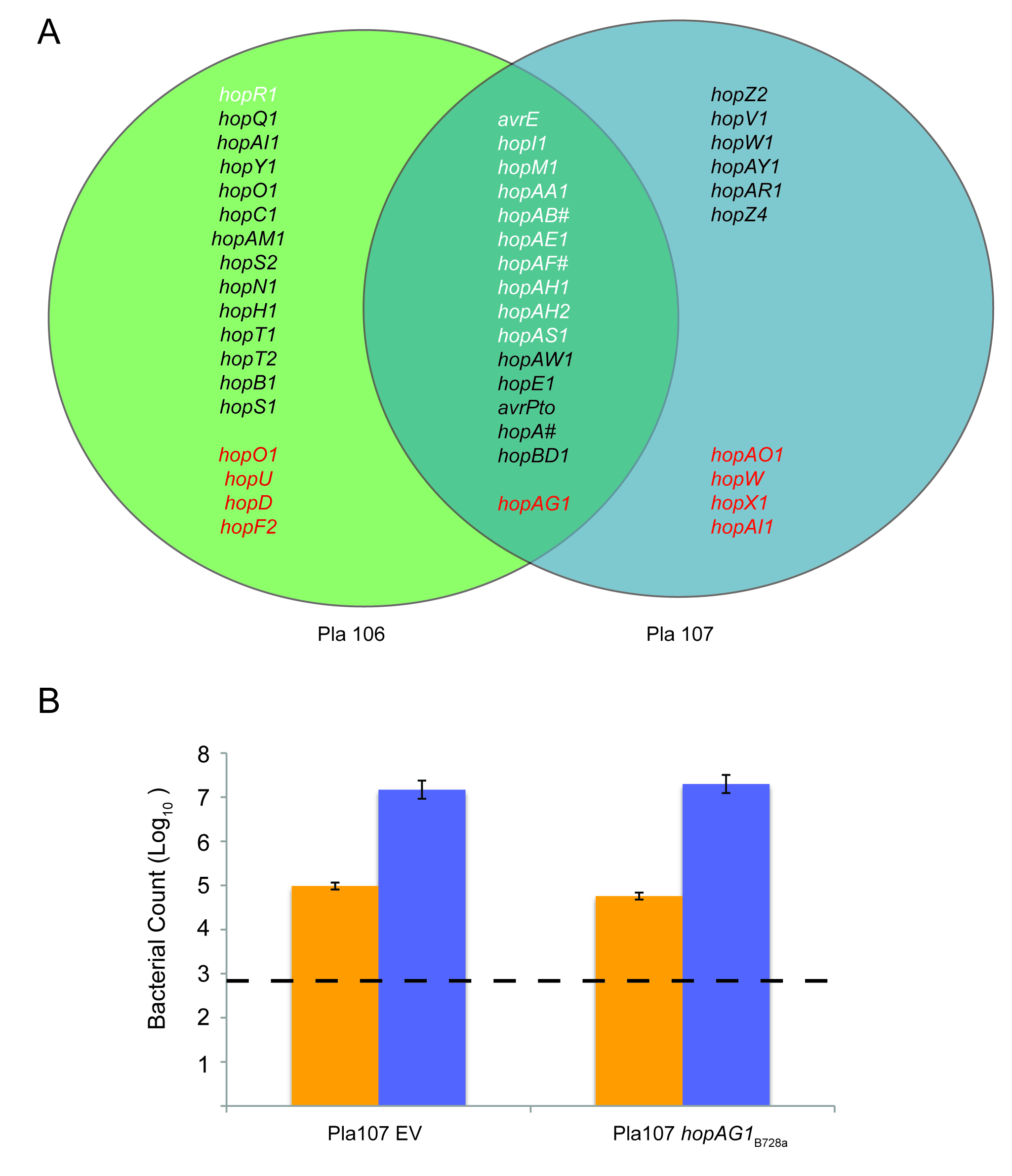

Supplement: Figure S15 — Distantly related P. syringae pv. lachrymans strains carry divergent TTE repertoires. (A) TTE repertoires were compared for Pla 106 and Pla 107, which are both isolated from diseased cucumbers but are members of different P. syringae phylogenetic groups. TTE families found within a majority (>10) of all sequenced P. syringae strains are shown in white, while those found in <10 genomes are listed in black. TTE disrupted by either truncation or insertion elements are listed in red. (B) A representative growth curve from two independent experiments (3 replicates for each genotype) on Cucumis sativus cv. Eureka is shown. Orange bars represent bacterial counts in inoculum while blue bars represent bacterial counts after 6 days of growth in planta. Dashed line represents estimated bacterial count in planta at day 0. Error bars indicate 1 standard error. (TIF) [file ppat.1002132.s024.tif]

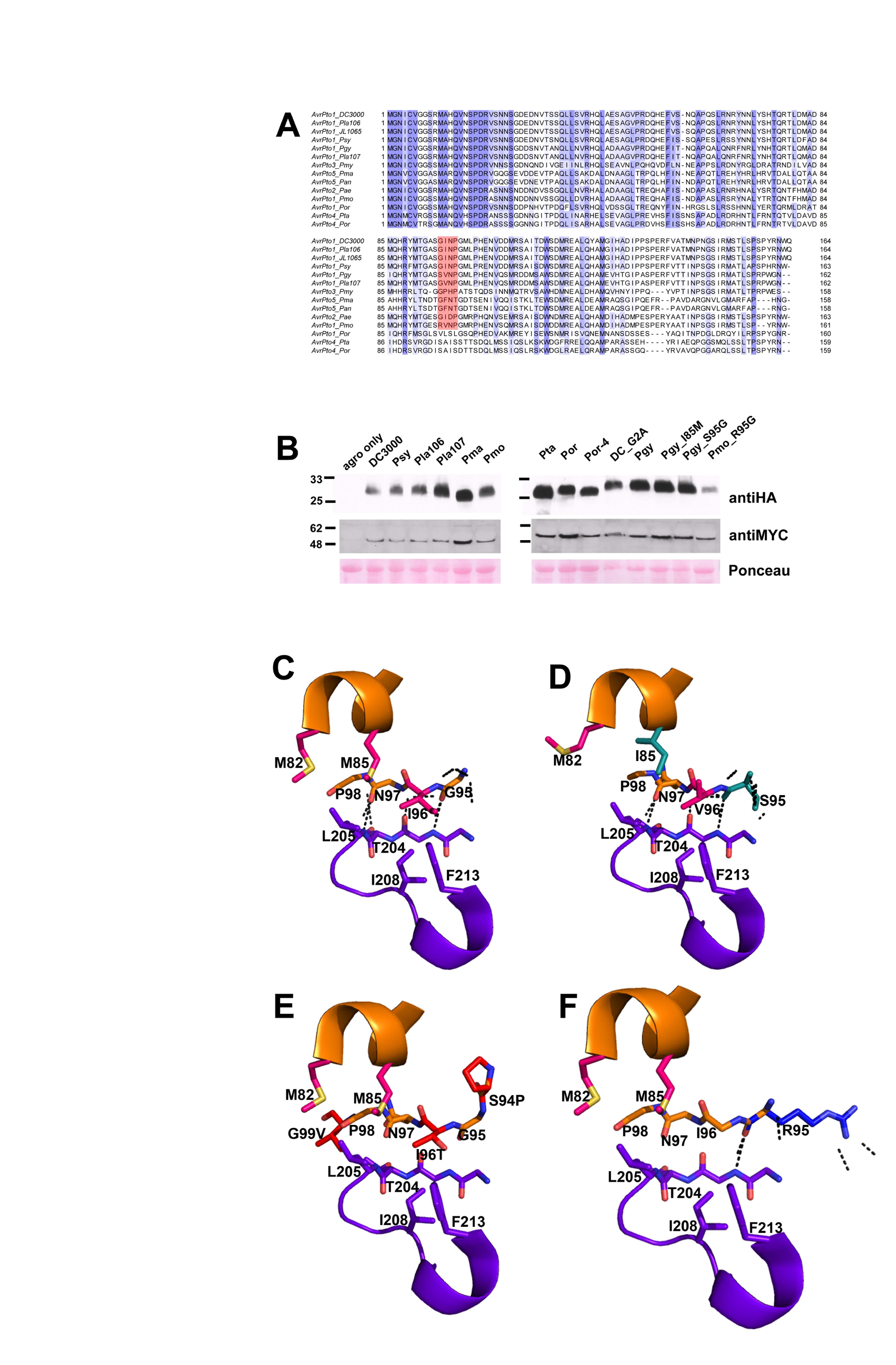

Supplement: Figure S16 — AvrPto alignment; expression of AvrPto/Pto in transient assays; GINP loop diversity. (A) ClustalW alignment of the AvrPto superfamily. Conserved residues are highlighted in blue. The GINP loop is highlighted in red. (B) Western blotting of Agrobacterium-N. benthamiana transient assay. AvrPto orthologs are HA epitope tagged, while Pto is tagged with the c-myc epitope. Ponceau staining reflects overall protein loading. Unrecognized AvrPto orthologs are expressed at least as well as AvrPto, indicating that lack of avirulence is not merely due to a lack of expression. (C) GINP loop region of the AvrPto/Pto co-crystal, orientation as in [58]. AvrPto is in orange, Pto purple. (D) AvrPtoPgy R4 sequence modeled onto the AvrPto crystal structure, I85 and G95 are shown in cyan. (E) AvrPto crystal structure with known loss of avirulence mutations S94P, I96T and G99V shown in red [64]. (F) AvrPtoPmo sequence modeled onto AvrPto crystal structure, S95 shown in blue. Images (C)–(F) generated using the PyMOL software package [98]. (TIF) [file ppat.1002132.s025.tif]

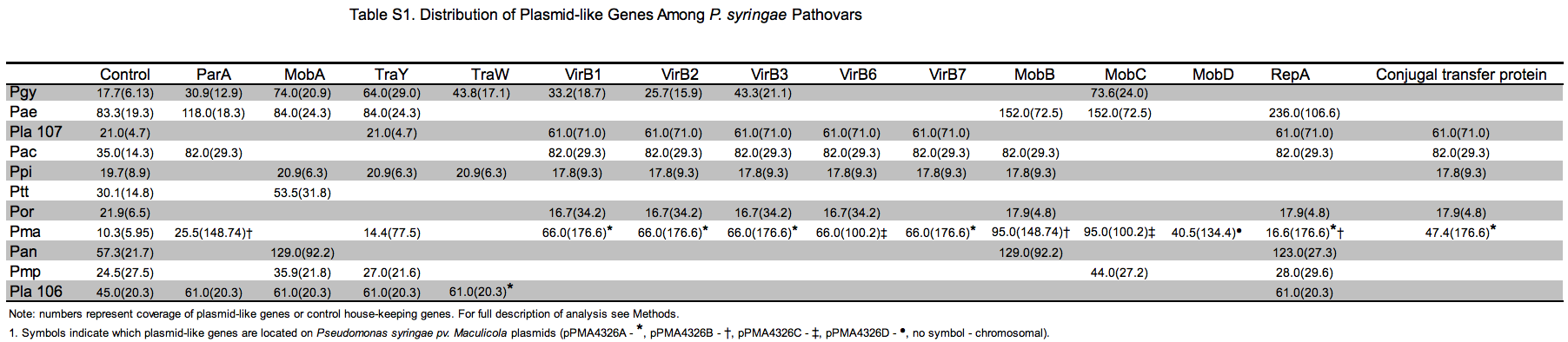

Supplement: Table S1 — A majority of P. syringae strains harbor endogenous plasmids. Putative plasmid sequences were identified through BLAST searches against fourteen common and typically plasmid localized sequences (horizontal). For each draft genome that contains putative plasmids (vertical), the average coverage level and standard deviation of coverage (in parentheses) over the contig containing that fragment is reported. As a control, the average coverage over gyrB, gapA, and rpoD is reported as an estimate for chromosomal coverage levels. Genomic data for Pmp has the highest difference between the average chromosome coverage (24.5) and putative plasmid sequence coverage (359.0 - MobA). (TIF) [file ppat.1002132.s026.tif]

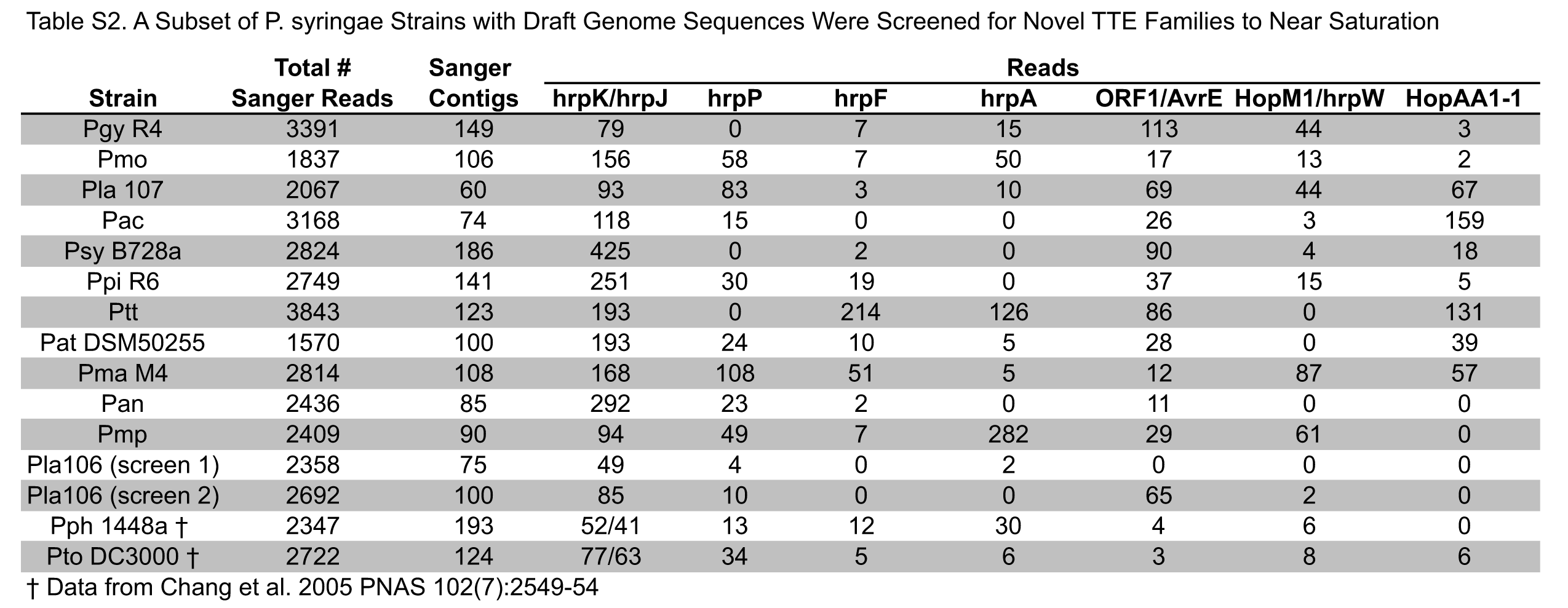

Supplement: Table S2 — A subset of P. syringae strains with draft genome sequences were screened for novel TTE families to near saturation. The number of contigs containing hrpL-regulated TTSS and common effector genes recovered in our functional screen is given, as are data for strains from the previously reported screen [23]. (TIF) [file ppat.1002132.s027.tif]

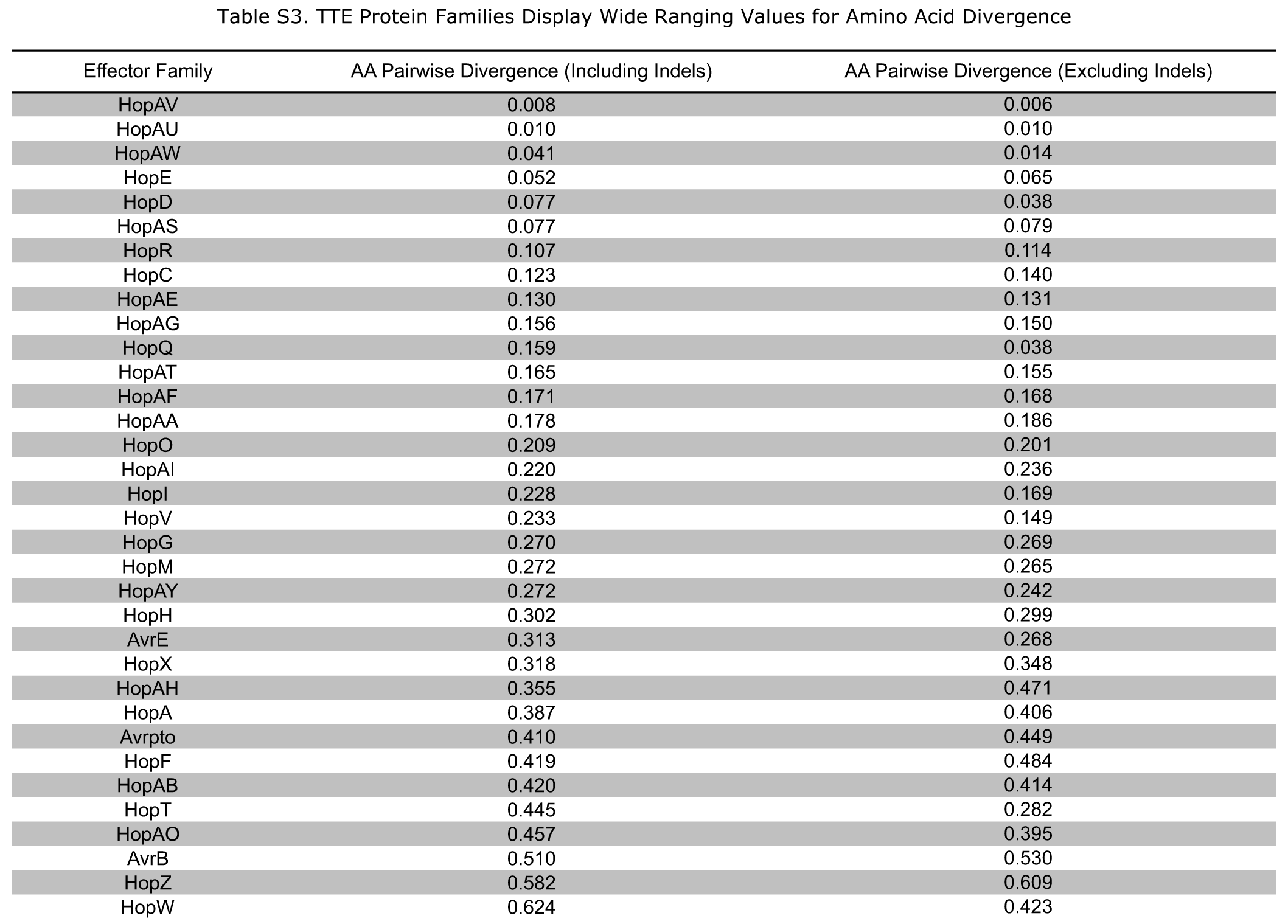

Supplement: Table S3 — TTE protein families display wide ranging values for amino acid divergence. Pairwise amino acid divergence was calculated for all TTE families represented in a majority of strains. Divergence was calculated both by including each positional gap in the sequence as a divergent site or by excluding gaps altogether. (TIF) [file ppat.1002132.s028.tif]
